# AQP8 promotes glioma proliferation and growth, possibly through the ROS/PTEN/AKT signaling pathway

**DOI:** 10.1186/s12885-023-11025-8

**Published:** 2023-06-06

**Authors:** Zhang Hao, Sheng Huajun, Guo Zhen, Xing Yu, Liu Qian, Cai Ziling, Shen Zihao, Xia Qingqian, Zhu Shujuan

**Affiliations:** 1 Department of Human Anatomy, College of Basic Medicine, Chongqing Medical University, Chongqing, 400016 China; 2 Department of Forensic Medicine, Chongqing Medical University, Chongqing, 400016 China

**Keywords:** Apoptosis, aquaporin-8, Glioma, Proliferation, ROS/PTEN/AKT pathway

## Abstract

**Background:**

The aquaporin (AQP) family of proteins has been implicated in the proliferation and growth of gliomas. Expression of AQP8 is higher in human glioma tissues than in normal brain tissues and is positively correlated with the pathological grade of glioma, suggesting that this protein is also involved in the proliferation and growth of glioma. However, the mechanism by which AQP8 promotes the proliferation and growth of glioma remains unclear. This study aimed to investigate the mechanism and role of abnormal AQP8 expression in glioma development.

**Methods:**

The dCas9-SAM and CRISPR/Cas9 techniques were used to construct viruses with overexpressed and knocked down AQP8, respectively, and infect A172 and U251 cell lines. The effects of AQP8 on the proliferation and growth of glioma and its mechanism *via* the intracellular reactive oxygen species (ROS) level were observed using cell clone, transwell, flow cytometry, Hoechst, western blotting, immunofluorescence, and real-time quantitative polymerase chain reaction assays. A nude mouse tumor model was also established.

**Results:**

Overexpression of AQP8 resulted in an increased number of cell clones and cell proliferation, enhanced cell invasion and migration, decreased apoptosis and phosphatase and tensin homolog (PTEN) expression, and increased phosphorylated serine/threonine protein kinase (p-AKT) expression and ROS level, whereas the AQP8 knockdown groups showed opposite results. In the animal experiments, the AQP8 overexpression group had higher tumor volume and weight, whereas the AQP8 knockdown group had lower tumor volume and weight compared with those parameters measured in the control group.

**Conclusions:**

Our results preliminary suggest that AQP8 overexpression alters the ROS/PTEN/AKT signaling pathway, promoting the proliferation, migration, and invasion of gliomas. Therefore, AQP8 may be a potential therapeutic target in gliomas.

**Supplementary Information:**

The online version contains supplementary material available at 10.1186/s12885-023-11025-8.

## Background

Glioma is the most common primary intracranial tumor and one of the most diverse and complex cancers in humans [1]. Surgical resection of tumor tissue and chemotherapy cannot achieve the ideal therapeutic effect because of glioma cells’ rapid proliferation and high invasiveness. Gliomas have a very high recurrence rate; therefore, the average life expectancy in patients with glioma is less than 15 months [2]. Given that water accounts for 80% of the brain volume, glioma often leads to brain edema and hernia, further increasing the risk of mortality. Aquaporins (AQPs), also called water channels, form a 13-member (AQP0–12) transmembrane protein family. They can transport water quickly because their distinct protein structure enables them to form pores in cell membranes.

This rapid transport of water by AQPs is a key mechanism in the development of glioma [3, 4]. Studies have shown that gliomas maintain their survival and proliferation through some highly expressed AQPs [5]. For example, AQP1 contributes to the formation of new blood vessels around gliomas, accelerating invasion toward surrounding brain tissues [6]. Specifically, AQP1 can increase the proportion of glioma-associated microglia and macrophage infiltration. Thereafter, it causes microglia to secrete more interleukin-6 (IL-6), which can result in binding glioma stem cells and their cell membrane receptors together, promoting the proliferation and growth of glioma stem cells through the IL-6/gp130/STAT3 pathway [7–10].

Furthermore, AQP4 plays a key role in the blood-brain barrier destruction in glioma [11], whereas AQP5 is closely related to glioma-related brain edema [12]. AQP9 participates in the oxidative phosphorylation process of cellular mitochondria in glioma and can accelerate the clearance of metabolic wastes such as glycerol and lactic acid outside glioma cells, which is conducive to the rapid proliferation of glioma cells [13, 14]. The synergistic effects of these AQPs make the tumor microenvironment conducive to the growth of glioma cells and resist the effect of chemotherapy in clinical treatment [15].

Another important member of the AQP family, AQP8, is more frequently investigated in cancers of the reproductive and digestive systems [16, 17], and less in those of the nervous system. However, previous studies have shown that the AQP8 expression level is abnormally high in human gliomas, especially glioblastoma. This high expression is positively correlated with the pathological grade of gliomas, suggesting that AQP8 may also be involved in promoting the proliferation and growth of gliomas [18]. However, the mechanism by which AQP8 exerts these effects remains unknown. Krüger et al. found that AQP8 participates in the transmembrane transport of H_2_O_2_ in RINm5F cells [19], and the concentration of H_2_O_2_ is a key factor affecting reactive oxygen species (ROS) level, which can regulate the growth state of cells. To determine if AQP8 plays a role in the maintenance of redox levels in glioma cell growth, this study aimed to investigate the role of abnormal AQP8 expression in the development of gliomas and its regulation by the ROS/PTEN/AKT signaling pathway. Toward this goal, knockdown and overexpression lentiviruses targeting AQP8 were constructed using CRISPR/Cas9 gene-editing technology and dCas9-SAM technology, respectively. Glioma cell lines were then infected with the viruses, and the effect and mechanism of AQP8 on the proliferation and growth of glioma were investigated. The findings of this study may provide new insights into the diagnosis and treatment of glioma.

## Methods

### Cell culture of experimental groups

The A172 and U251 human glioma cell lines were purchased from the Chinese Academy of Sciences Cell Bank (Shanghai, China). Cells were cultured in Dulbecco’s Modified Eagle Medium (DMEM; HyClone, GE Healthcare Life Sciences, Logan, UT, USA) supplemented with 10% fetal bovine serum (FBS, HyClone; GE Healthcare Life Sciences, USA), 100 U/mL penicillin-streptomycin (HyClone; GE Healthcare Life Sciences), and 2 mM glutamine (HyClone; GE Healthcare Life Sciences). Cells were cultured at 37 °C in a humidified atmosphere containing 5% CO_2_. AQP8 was detected by western blot (see details below). The cells were divided into four groups: (1) control group (no virus infection); (2) negative control group (infected with negative control lentiviruses and sgRNA, NC); (3) knockdown group (infected with lentiviruses containing Cas9 protein and corresponding sgRNA, K.D); and (4) overexpression group (infected with lentiviruses containing dCas9 protein and corresponding sgRNA, O.E). (Appended part: on the basis of the original 4 groups, the overexpression group was supplemented with empty vector control as its negative control group to show that the negative control group had no significant effect on the growth viability of the cells after infection of two glioma cell lines through CCK-8 and MTT experiments. The specific experimental methods and results of this part of the supplementary data can be found in: https://pan.baidu.com/s/1idLoUOclI8tivHg2ThImSw?pwd=j476)

### Virus construction and cell infection

The sgRNA interference target sequence of the *AQP8* gene was designed containing the adhesive end of the restriction site, and the single-stranded DNA oligo was synthesized and purified by polyacrylamide gel electrophoresis (PAGE). Double-stranded DNA was formed by annealing and cloned into the Lenti-SgRNA-tagvectorGV371 (U6-SGRNA-SV40-EGFP). TOP10 receptive state transformation was performed, and colony polymerase chain reaction (PCR) was used to detect positive clones. Clones with lentivirus knockdown were verified for correct expression after sequencing with the following primers: lentivirus sgRNA sequence knockdown 1: 5′-CTGCACAAACCGTTCGTACC-3′, sgRNA sequence knockdown 2: 5′-TGGTGATGCTCCTCCCGTAC-3′, and sgRNA sequence knockdown 3: 5′-TCCCATTCTCAATGACCACC-3′. Next, AQP8 was overexpressed by lentivirus vector GV468 (U6-SGRNA-SV40-MS2-P65-HSF1-CMV-EGFP) in the SAM system and confirmed using primers sgRNA sequence overexpression 1: 5′-GCGGCTCTGAGGCCCAGAAG-3′, sgRNA sequence overexpression 2: 5′-TGCTGAACTTTCCGCCAGTG-3′, and sgRNA sequence overexpression 3: 5′-TTTTTAAATCTCAACAGGGC-3′.

The design and packaging of the lentivirus and the determination of the virus titer were completed by GeneChem LTD (GeneChem, Shanghai, China). A172 and U251 cells were inoculated into six-well plates at 1 × 10^6^ cells/well and grown to 35 − 50% confluency. A172 cells and U251 cells were infected with negative control Cas9 and dCas9 lentivirus, respectively, following manufacturer’s instructions. After 3 days of routine culture, uninfected cells were removed by puromycin selection. After another 3 days of culture, the cells were again infected with lentivirus with sgRNA. After 48 h of cell infection, cells in each group were collected to detect green fluorescent protein (GFP) expression, and those with an infection rate of more than 85% were selected for subsequent tests.

### Real-time PCR

The RNA was extracted from each group of cells using the RNAiso Plus Kit (Takara, Kusatsu, Japan), and the concentration was measured. The RNA was converted into first-strand cDNA according to manufacturer’s instructions. PCR amplification was performed using the LightCycler system with the amplification kit. The primers were: AQP8 upstream 5′-TGCCATCAATGAGAAGACAAAG-3′, AQP8 downstream 5′-ATCTCCAATGAAGCACCTAATG-3′; β-actin was used as the internal reference gene: β-actin upstream: 5′ -AGAAAATCTGGCACCACACCT-3′ and downstream: 5′-GATAGCACAGCCTGGATAGCA-3′. The amplification cycles were as follows: 95 °C for 5 min, 95 °C for 30 s, 57 °C for 30 s, and 72 °C for 45 s, total 40 cycles. The data were analyzed using the 2-ΔΔCt method. All viruses used in the follow-up studies, except those in the negative control group, produced the highest overexpression/knockdown as verified by real-time quantitative PCR.

### Cell proliferation experiments

#### Colony formation

The A172 and U251 cells were collected from each group, inoculated in a six-well plate at 500 cells/well, and cultured in a complete medium containing 30% FBS. The medium was changed every two days for 12 days. The cells were washed with phosphate-buffered saline (PBS) and fixed with 4% paraformaldehyde at room temperature for 10 min. The cells were washed with PBS and stained with crystal violet (Beyotime, Shanghai, China) at room temperature for 10–15 min. Cells in each six-well plate were observed with a microscope, and the number of cell clones was counted. The standard record of ≥ 50 cells in each group was taken as the effective number of clones. Six-well plates were photographed using A4 paper as a background (Leica, Wetzlar, Germany).

#### 25-Ethynyl-2,-deoxyuridine incubation

The U251 and A172 cells were incubated in 10 µM 5-ethyl-2-deoxyuridine (EDU; Beyotime, Shanghai, China) for 2 h following the manufacturer’s instructions. The cells were rinsed with PBS and fixed with 4% paraformaldehyde. Thereafter, the cells were treated with 200 µL 1 Apollo reaction mixture for 30 min, stained with 4,6-diamino-2-phenylindole (DAPI) for 5 min, and observed under fluorescence microscopy (Leica Microsystems, Wetzlar, Germany).

### Apoptosis experiments

#### Flow cytometry

The A172 and U251 cells were obtained from each group, inoculated in six-well plates at 2 × 10^5^ cells/well, and cultured for 48 h. The cells were then rinsed with PBS and digested in trypsin. After digestion was terminated, the cell suspension was transferred to 15-mL centrifuge tubes and centrifuged at 800 rpm for 3 min. The supernatant was removed, and the cells were resuspended in PBS. Centrifugation was repeated, and the supernatant was removed again. This process was repeated twice. The cells from each group were then resuspended in 500 µL PBS and transferred into 1.5-mL Eppendorf (EP) tubes. Cellular apoptosis was measured using an Annexin V-Fluorescein Isothiocyanate and Propidium Iodide Apoptosis Detection Kit (BD Biosciences, Franklin Lakes, NJ, USA). Apoptosis rates were measured using FACSCalibur flow cytometry (BD Biosciences) according to the manufacturer’s instructions. Apoptosis rate = Up Right(UR) + Lower Right(LR).

#### Hoechst staining

The A172 and U251 cells from each group were inoculated in six-well plates at 5 × 10^4^ cells/well and fixed with 4% paraformaldehyde for 10 min. The cells were then washed with PBS, stained with Hoechst solution (Hoechst, Beyotime) for 10 min, rinsed thrice with PBS, and observed using fluorescence microscopy (Leica Microsystems).

#### Western blot

Cells in each group were collected in T25 bottles, and radioimmunoprecipitation assay buffer (RIPA) with phenylmethylsulfonyl fluoride (PMSF) (RIPA: PMSF = 100:1) was added. Cells were collected using a disposable cell scraper, and the lysate was transferred to 1.5-mL EP tubes. RIPA solution was added to adjust the final protein concentration of each group to 2.5 mg/mL. Sodium dodecyl sulfate (SDS)-protein loading buffer and lysate were then mixed in a 1:4 ratio and denatured at 95 °C for 5 min. Protein loading solution (12 µL) was added to each sample, and SDS-PAGE was performed at 80 V for 30 min and 120 V for 60 min. After the gel is cut near the corresponding molecular weight and displayed by the Marker, it was then placed into an ice box in preparation for electroblotting.

The separated proteins were transferred from the gel to a polyvinylidene fluoride (PVDF) membrane by electroblotting for 30 min at 250 mA, and the PVDF membrane was then sealed with rapid sealing solution (Beyotime) at room temperature for 20 min. The membrane was immersed once in Tris-buffered saline Tween solution, and the PVDF membrane was then incubated overnight on ice in the presence of the primary antibodies (AQP8 dilution ratio, 1:1,000; Abcam, Cambridge, UK) (β-actin, Bax, Bcl-2 dilution ratio, 1:1,000; AKT, p-AKT, PTEN dilution ratio, 1:2,000; Cell Signaling Technology, Danvers, MA, USA). Subsequently, the PVDF membrane was incubated in the presence of the secondary antibody (secondary antibody dilution ratio 1:5,000; Proteintech, Rosemont, IL, USA) for 1 h at 37 ℃ with constant shaking. Bands were detected using hypersensitive enhanced chemiluminescence color solution, and color imaging was performed using the Image Lab Software. The strips were preserved for analysis.

### Transwell experiment

#### Invasion experiment

Matrigel was refrigerated overnight at 4 °C. Matrix glue diluted with DMEM (100 µL) was added to the upper chamber of the transwell plate using a 200-µL precooled gun tip (diluted according to the manufacturer’s instructions, with the final concentration of the matrix glue at 2 mg/mL). The solution was then incubated overnight. Respectively, 500 µL A172 and U251 cells from each group were then added in the upper chamber of the transwell plate at 5.0 × 10^4^ cells/well, and cultured for 24 h. The cells were rinsed with PBS, and 500 µL 4% paraformaldehyde was added to the lower chamber for cell passage. The cells were rinsed again with PBS, and 500 µL crystal violet was added to the lower chamber for staining for 10 min. The cells were rinsed with PBS, and the upper chamber was gently wiped with cotton swabs to remove residual cells. Finally, the cells were dried and observed under fluorescence microscopy (Leica Microsystems).

#### Migration experiment

The steps were the same as for the invasion experiment, except that no matrix glue was added.

#### ROS production measurement

The cells in each group were inoculated into a six-well plate at 5 × 10^5^ cells/well. After conventional culture for 24 h, the intracellular ROS level of target cells was measured using a dihydroethidium (DHE) assay kit-ROS (ab236206, Abcam China, Shanghai, China). The cells were processed according to the manufacturer’s instructions, digested with trypsin, and centrifuged at 400 *g*. A 100 µL cell suspension was drawn from a 500 µL cell suspension and the cells were re-suspended in a 300 µL ROS buffer, and the fluorescence intensity was measured at 480 nm excitation wavelength and 570 nm emission wavelength.

#### Evaluation of oxidative stress

The levels of superoxide dismutase (SOD), malondialdehyde (MDA), and glutathione peroxidase (GSH-PX) in each group were determined using the SOD, MDA, and GSH-PX detection kits following the manufacturer’s instructions (Nanjing Bioengineering Institute, Nanjing, China).

#### In vivo mouse xenograft

Female BALB/C nude mice (n = 6) aged 4 − 6 weeks weighing 16 − 20 g were purchased from Beijing Vitong Lihua Biotechnology Co., Ltd. (Beijing, China). The U251 cells from the control, negative control, overexpression, and knockdown groups were implanted subcutaneously at 1 × 10^8^ cells per mouse. Tumor volume and weight of the nude mice were recorded at 3-day intervals beginning at 4 weeks after subcutaneous inoculation. Tumor volume was calculated as V = (length × width^2^)/2. After 20 days, the mice were intraperitoneally injected with pentobarbital solution at a dosage of 150 mg/kg for each nude mouse. After complete anesthesia, the mice were sacrificed by spinal dislocation, and the tumors were extracted and weighed for subsequent analysis. All animal studies were approved by the Animal Use and Care Committee of Chongqing Medical University. All applicable international, national, and/or institutional guidelines for the care and use of animals were followed.

### Statistical analysis

All experiments were performed in triplicate. Graphs were generated using GraphPad Prism, ver. 8.2 (GraphPad Software, San Diego, CA, USA, www.graphpad.com). ImageJ software was used to quantify the western blotting (WB) and PCR results. Data were presented as means ± standard deviation. One-way analysis of variance followed by Bonferroni’s test was used to compare differences among groups. Significance was expressed as *p < 0.05, **p < 0.01, or ***p < 0.001.

## Results

### Viruses that overexpressed and knocked down AQP8

**Fig. 1:**
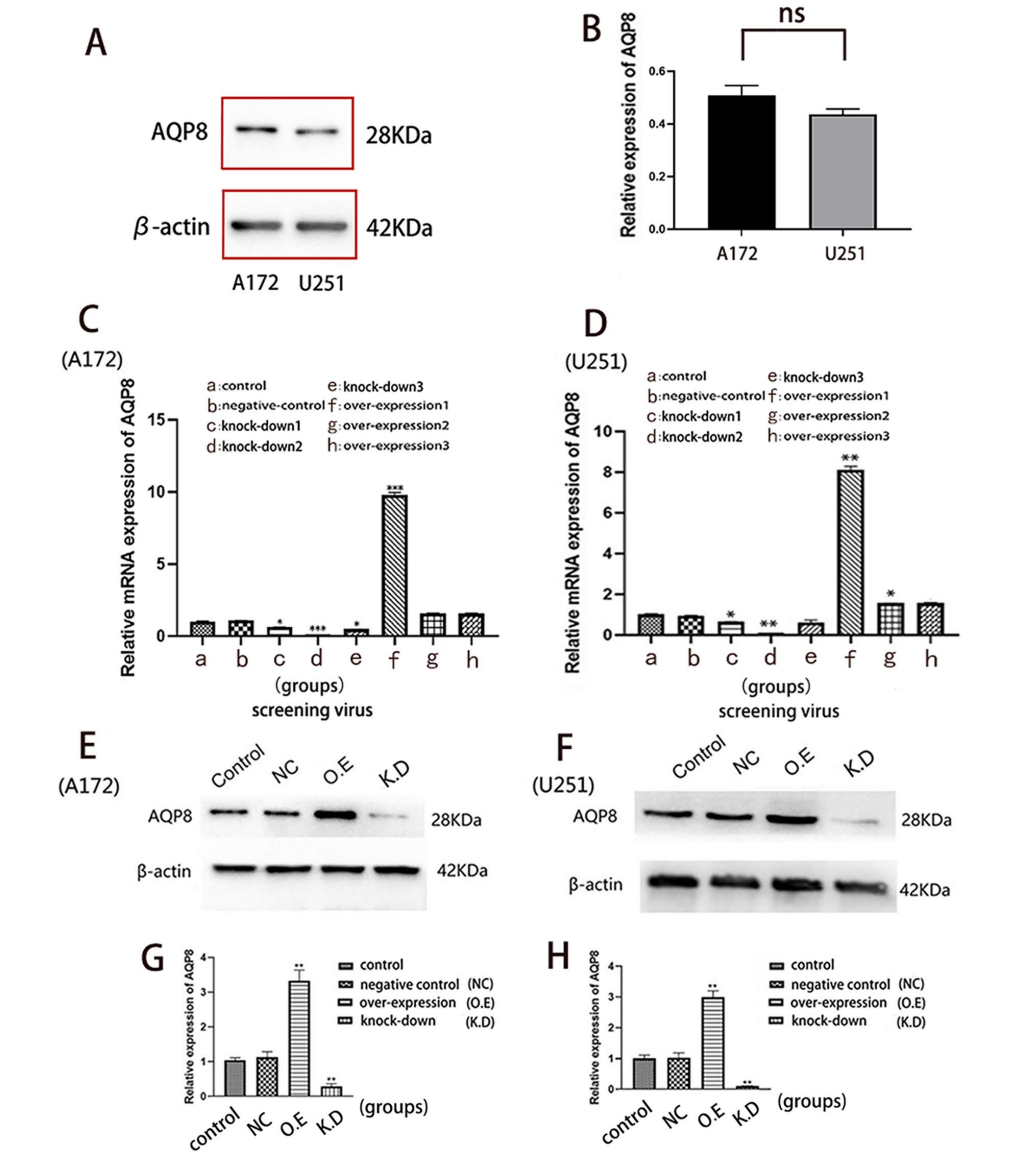
Screening and identification of viruses with overexpressed and knocked down AQP8. **A-B** Confirmation of the expression of AQP8 in A172 and U251: (**A**) The expression level of AQP8 in A172 and U251 cells. (**B**) The comparison of AQP8 protein content (ns, no significant difference). **C-D** Virus screening: (**C, D**) relative mRNA expression analysis of AQP8 in all groups, including one negative control, three overexpression and three knockdown groups of AQP8 (vs. control, *p < 0.05, **p < 0.01). Viruses d and f have better effects on overexpression and knockdown in both cell types. **E-H** NC virus screening for an overexpressed virus and a knockdown virus by western blot: **(E)** Expression of AQP8 in different groups after A172 cell infection. **(G)** Bar graph of the relative expression of AQP8 in A172 cells. **(E)** Expression of AQP8 in different groups after U251 cell infection. **(H)** Bar graph of the relative expression of AQP8 in U251 cells. AQP, aquaporin; NC, negative control; O.E, overexpressed; K.D, knockdown. All experiments were performed in triplicate

The results of WB indicated that there was no significant difference in the expression of AQP8 protein between A172 and U251 cells (Fig. 1A, B).

The results of the RT-qPCR showed that, first, the virus in the negative control group was effective. Second, the three constructed AQP8 overexpressed and knockdown bivectors were all effective, among which the knockdown group 2 and overexpression group 1 had more significant effects. Therefore, the viruses used in the subsequent infection experiments in this study were those in the negative control group, knockdown group 2, and overexpression group 1 (Fig. 1C, D). After infecting A172 and U251 cells, WB results showed that AQP8 expression was markedly higher in the overexpressed group and lower in the knockdown group than in the control group (Fig. 1E-H). This indicated that the virus was effective and could be used in subsequent experiments.

### Effects of AQP8 expression on proliferation, migration, and invasion of glioma cells

**Fig. 2:**
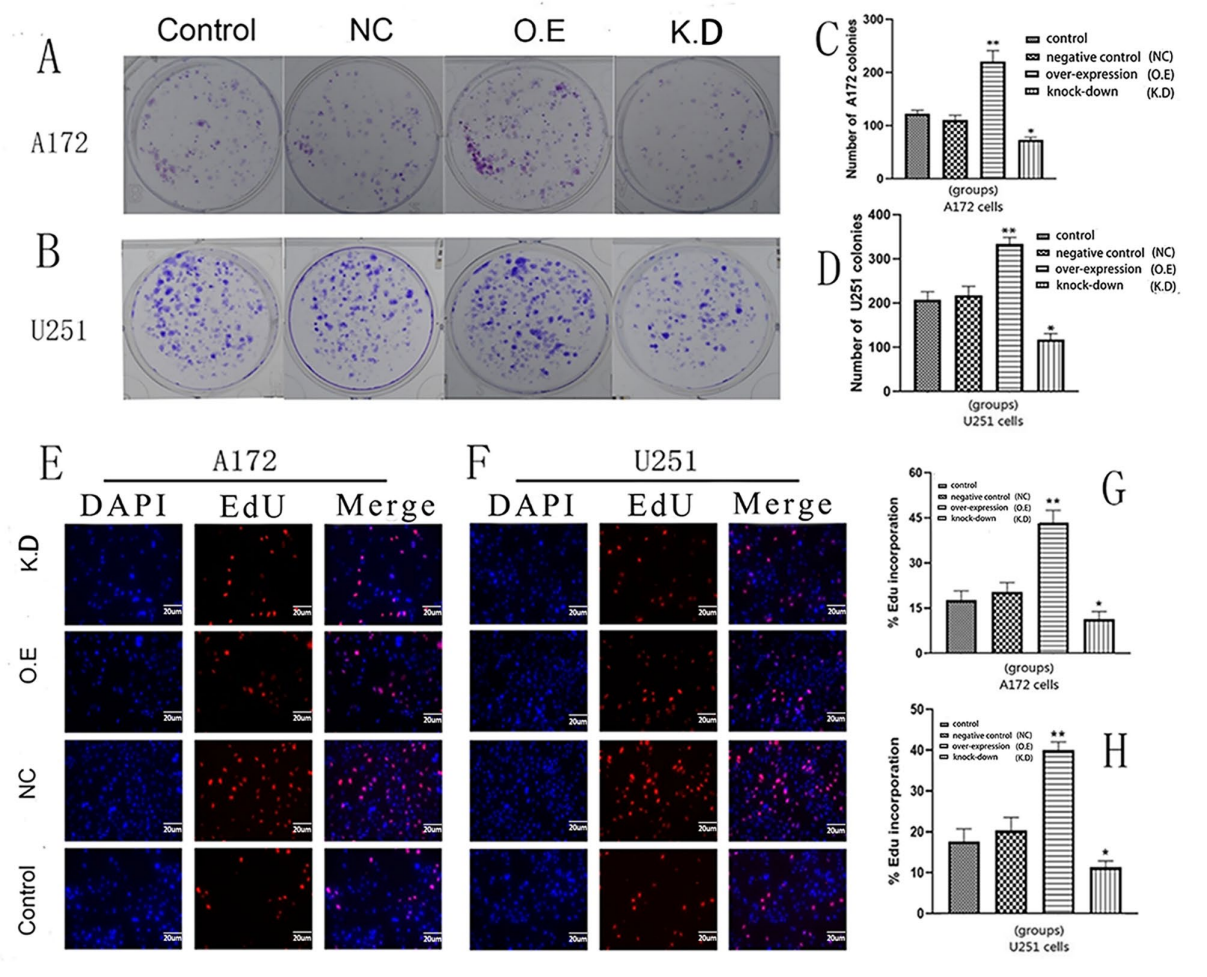
Cell clone and EDU experiments showing the proliferation capability of A172 and U251 cells infected by different viruses. **(A, B)** Cell clone experiment. The colony formation capability of A172 and U251 cells is increased by AQP8 overexpression (O.E) and decreased by AQP8 knockdown (K.D) compared with the control group. **(C, D)** Bar graphs of colony formation of A172 and U251 cells. Complete proliferation analysis was performed by observing the number of cell clones (vs. control, *p < 0.05, **p < 0.01). **(E, F)** EDU fluorescence staining experiment. Red represents EDU, and blue represents nucleus. Fluorescence intensity in A172 and U251 cells is increased by AQP8 overexpression and decreased by AQP8 knockdown. **(G, H)** EDU percentage and analysis in A172 and U251 cells of different groups (vs. control, *p < 0.05, **p < 0.01). EDU, 5-ethyl-2-deoxyuridine; AQP, aquaporin; NC, negative control; O.E, AQP8-overexpressed; K.D, AQP8-knockdown; DAPI, 4,6-diamino-2-phenylindole. All experiments were performed in triplicate

**Fig. 3:**
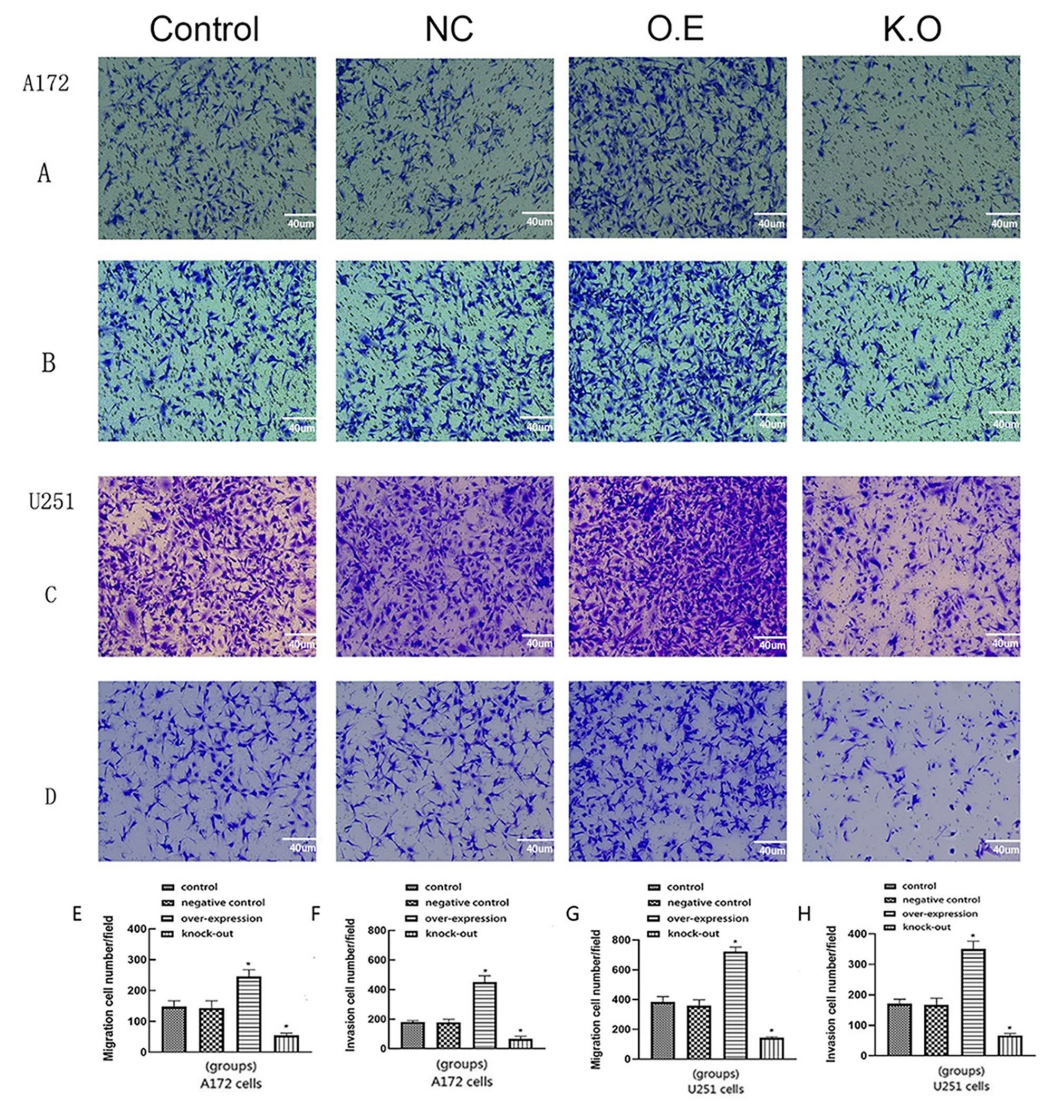
Migration and invasion capabilities of A172 and U251 cells that were infected by different viruses in the transwell experiment. **(A, C)** Cell migration phenomenon in all groups. **(B, D)** Cell invasion performance in all groups. **(E, F)** Migration and invasion numbers analysis of A172 cells in each group. Migration and invasion capabilities of A172 cells are enhanced by AQP8 overexpression and reduced by AQP8 knockdown. **(G, H)** Migration and invasion numbers analysis of U251 cells in each group. Migration and invasion capabilities of U251 cells were enhanced by AQP8 overexpression (O.E) and reduced by AQP8 knockdown (K.D) compared with control group (vs. control, *p < 0.05). AQP, aquaporin; NC, negative control; O.E, overexpressed; K.D, knockdown. All experiments were performed in triplicate

Cell cloning experiments showed that the number of A172 and U251 cells increased in the AQP8 overexpression group, whereas it significantly decreased in the AQP8 knockdown group (Fig. 2A–D). The EDU experiment showed that cell proliferation increased in the AQP8 overexpression group, whereas it significantly decreased in the AQP8 knockdown group (Fig. 2E–H). The results of the transwell assay showed that, compared with the control group, the AQP8 overexpression group had enhanced invasion and migration capabilities of A172 and U251 cells, whereas the knockdown group had significantly weakened the corresponding capabilities of these cells (Fig. 3). In the mouse model, compared with the control group, the AQP8 overexpression group had larger tumor volume and weight, whereas the AQP8 knockdown group had smaller tumor volume and weight. This indicated that AQP8 affected the proliferation and growth of gliomas (Fig. 7). Specifically, high AQP8 expression promoted the proliferation and growth of glioma to some extent, while inhibition of AQP8 expression slowed down these activities.

### Effects of AQP8 expression on apoptosis of glioma cells

**Fig. 4:**
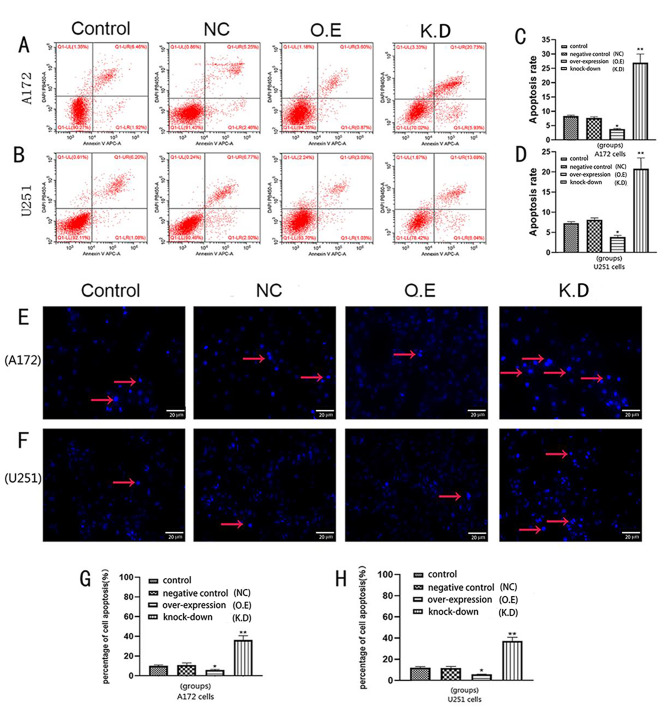
Effect of AQP8 overexpression and knockdown on apoptosis in A172 and U251 cells. **(A-D)** Apoptosis rates of A172 and U251 cells in each group on flow cytometry of PI- and annexin V-stained cells. AQP8 overexpression (O.E) and knockdown (K.D) reduced and increased the apoptosis rate in A172 and U251 cells, respectively (vs. control, *p < 0.05, **p < 0.01). **(E-F)** Hoechst staining on A172 and U251 cells of each group. Red arrows in E and F refer to apoptotic cells (blue). **(G, H)** The percentage of apoptotic A172 and U251 cells in each group with Hoechst staining. (vs. control, *p < 0.05, **p < 0.01). AQP, aquaporin; NC, negative control; O.E, overexpressed; K.D, knockdown. All experiments were performed in triplicate

Flow cytometry showed that, compared with the control group, the AQP8 overexpression group had a slightly lower apoptosis rate in A172 and U251 cells, while the AQP8 knockdown group had significantly higher apoptosis rates in A172 and U251 cells, especially in the early stage (Fig. 4A–D). Hoechst staining results also showed that the apoptosis rate was decreased in the overexpression group and significantly increased in the knockdown group (Fig. 4E–H). The WB results showed that Bcl-2 expression was higher while Bax expression was lower in the AQP8 overexpression group than in the control group and negative control group. Meanwhile, Bcl-2 expression was lower in the knockdown group (Fig. 6D–E, I-J). These results suggest that high AQP8 expression inhibits the apoptosis of glioma cells, and the inhibition of AQP8 expression promotes apoptosis.

### Effects of AQP8 expression on the redox state

**Fig. 5:**
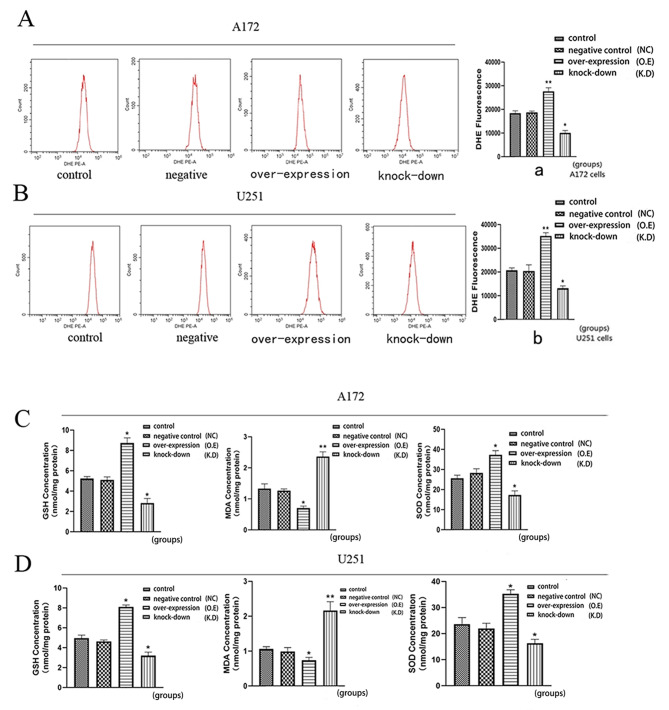
Determination of the intracellular redox environment of A172 and U251 cells. **(A, B, a, b)** DHE method for evaluating ROS production in A172 and U251 cells in each group. **(C, D)** Changes in GSH-PX, MDA, and SOD expression of A172 and U251 cells in each group (vs. control, *p < 0.05, **p < 0.01). DHE, dihydroethidium; ROS, reactive oxygen species; GSH-PX, glutathione peroxidase; MDA, malondialdehyde; SOD, superoxide dismutase; NC, negative control; O.E, overexpressed; K.D, knockdown. All experiments were performed in triplicate

The DHE results showed that, compared with the control group, the AQP8 overexpression group had higher ROS production, whereas the AQP8 knockdown group had lower ROS production (Fig. 5A, B, a, b). In the AQP8 overexpression group, the GSH-PX and SOD levels were increased, whereas those of MDA were decreased. The opposite results were obtained in the AQP8 knockdown group. The trends of expression of the two cell types were consistent (Fig. 5C, D). Concerning the MDA level, a high redox level after AQP8 overexpression did not damage glioma cells, but it decreased the level of lipid peroxides by increasing the levels of SOD and GSH. Meanwhile, AQP8 knockdown reduced SOD and GSH-PX levels and increased MDA levels. This indicates that changes in AQP8 expression affect intracellular ROS production and the redox balance.

### Effects of AQP8 expression on PTEN/AKT phosphorylation and glioma cell proliferation and growth

**Fig. 6:**
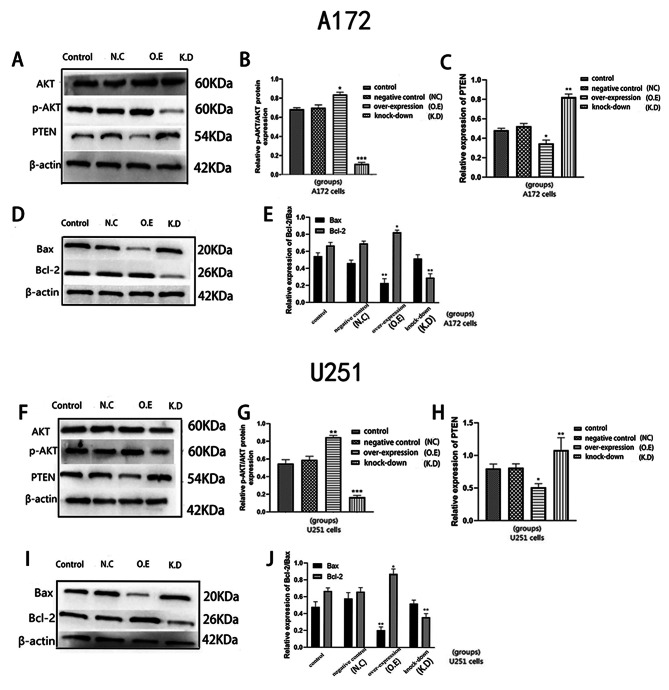
Western blot staining results. **(A-E)** A172; **(F-J)** U251. **(A,D,F,I)** Western blot showing the expression levels of PTEN, AKT, p-AKT, Bax, and Bcl-2 proteins in A172 and U251 cells. **(B,C,E,G,H,J)** Analysis of the effect of AQP8 on these proteins (vs. control, *p < 0.05, **p < 0.01, ***p < 0.001). AQP, aquaporin; PTEN, phosphatase and tensin homolog; AKT, serine/threonine protein kinase; p-AKT, phosphorylated AKT; NC, negative control; O.E, overexpressed; K.D, knockdown. All experiments were performed in triplicate

Phosphatase and tensin homolog (PTEN) and serine/threonine protein kinase (AKT) phosphorylation are important downstream regulators of the ROS signal transduction pathway. The WB results showed that the changes in PTEN and phosphorylated AKT (p-AKT) proteins in A172 and U251 cells were consistent. Compared with the control group, the AQP8 overexpression group had lower PTEN expression and higher p-AKT expression, whereas the AQP8 knockdown group had lower p-AKT expression (Fig. 6A–C, F–H). This indicates that AQP8 knockdown increases PTEN expression and decreases AKT phosphorylation.

**Fig. 7:**
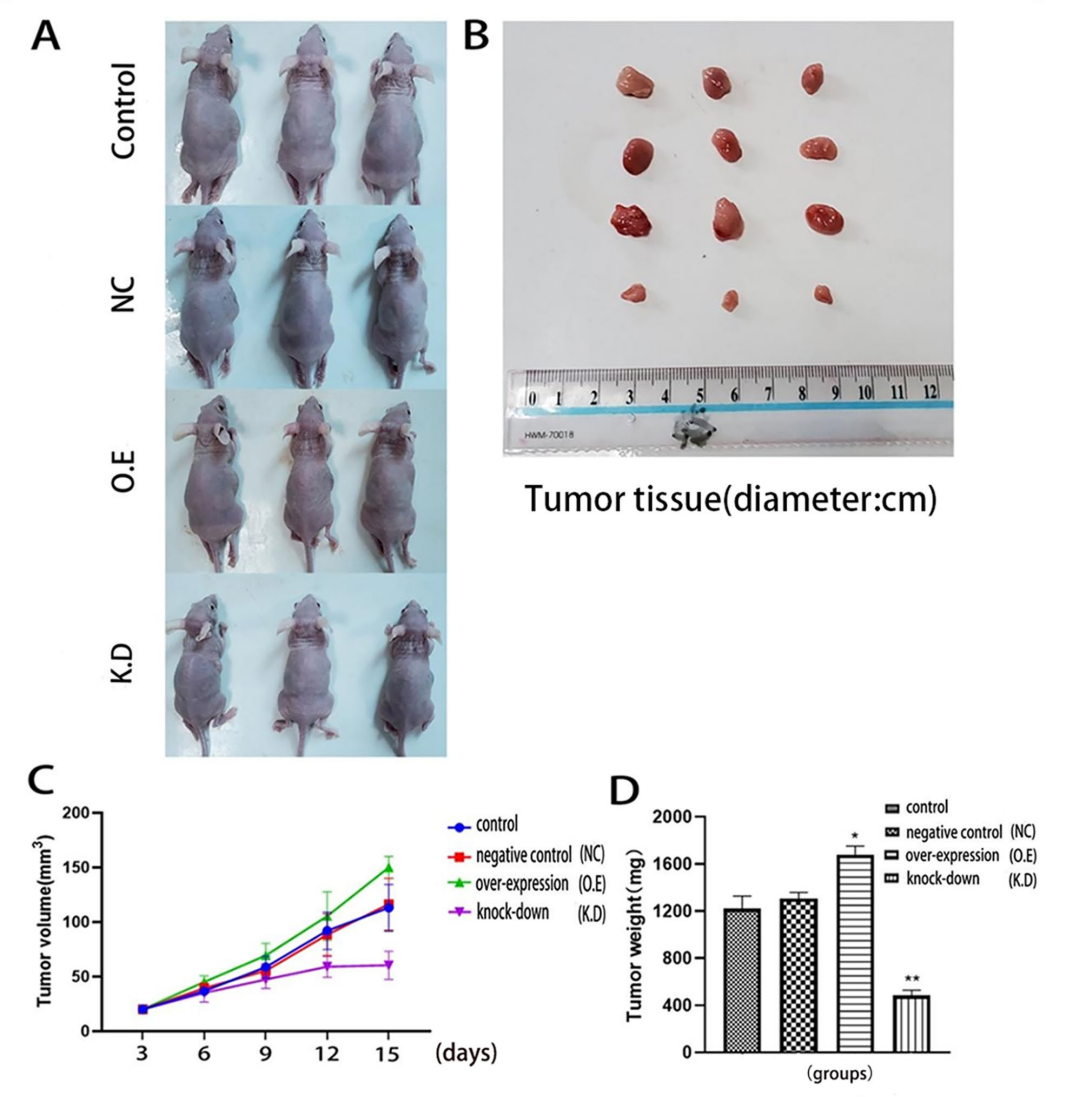
AQP8 affects tumor growth in vivo in a nude mouse tumor model injected with three groups of cells: NC, O.E, and K.D. **(A)** Nude mice in each group. **(B)** Representative images of xenograft tumors derived from the four groups of U251 cells. **(C)** Growth curves of U251 cell-derived tumors. **(D)** Weights of U251 cell-derived tumors. Compared with the control group, the growth of tissues in O.E group was faster, but that in K.D group was slower (vs. control, *p < 0.05, **p < 0.01). AQP, aquaporin; NC, negative control; O.E, overexpressed; K.D, knockdown. (n = 10; 4–6 weeks old.)

## Discussion

Previously, AQPs have been confirmed to affect the development of glioma [20]. For example, AQP1 is involved in the formation of peritumoral neovascularization of glioma [6]. Meanwhile, AQP4 affects the invasion and migration of glioma and is closely related to vasogenic edema and cytotoxic edema induced by glioma [11, 21]. The epidermal growth factor receptor/extracellular signal-regulated kinase/p38/mitogen-activated protein kinase (EGFR/ERK/p38 MAPK) signaling pathway affects glioma proliferation and apoptosis [12], and AQP9 is involved in the oxidative phosphorylation pathway in glioma cells [13]. These studies indicate AQPs participate in many aspects of glioma development. However, different types of AQPs play different roles in gliomas. Thus, further in-depth studies of other members of the AQP family will clarify the role of AQPs in the proliferation and growth of gliomas and provide further evidence for targeted therapy.

As a member of the AQPs, AQP8 has attracted attention because of its involvement in the development of many tumors, including ovarian and esophageal carcinomas [22, 23]. However, there have been few studies on its role in glioma. Originally, we detected the expression level of AQP8 protein in three cell lines, U87, A172 and U251. However, by using genetic profiling and transcriptome analysis in human glioma cell lines, Allen et al. [24] found that the DNA profile of the widely used glioma cell line U87MG was different from that of the original cells and that it is likely to be a bona fide human glioblastoma cell line of unknown origin. Therefore, only A172 and U251 were selected for our subsequent experiments and the results which contained three protein bands were cut, and the expression results of A172 and U251 were retained as Fig. 1A.

APQ8 loss-of-function was achieved by CRISPR-Cas9 mediated knockout in this study. However, in this context, the remaining APQ8 protein expression (Fig. 1C-H) is potentially concerning. This was due to the fact that the target cells which infected by lentivirus did not be screened and the monoclonal cells did not be cultured.Considering that a small number of cells still expressed AQP8, which however does not affect the conclusion of the whole experiment to some extent. AQP8 activation and knockdown in glioma A172 and U251 cells showed that, to some extent, AQP8 knockdown reduced the proliferation, migration, and invasion capabilities of glioma cells. The AQP8 overexpression in glioma cells increased proliferation, migration, and invasion. Tumorigenesis experiments in nude mice also showed that AQP8 overexpression enhanced tumor growth, whereas AQP8 knockdown had the opposite effect. Collectively, these results showed that high AQP8 expression promotes the proliferation and growth of glioma. The results of flow cytometry and the Hoechst assay both supported that glioma cells overexpressing AQP8 had lower early apoptosis rates, whereas those with AQP8 knockdown had significantly higher early apoptosis rates.

Bcl-2 and Bax belong to the same family, and the heterodimer formed by Bcl-2 and Bax reduces the permeability of the mitochondrial membrane and prevents apoptosis [25, 26]. Further, the ratio of Bcl-2 to Bax is one of the indicators of cell apoptosis capability [27]. The WB analysis in this study showed that Bcl-2 expression was significantly decreased in AQP8 knockdown glioma cells, whereas Bcl-2 expression was increased and Bax expression was decreased in AQP8 overexpressing glioma cells. Further, the Bcl-2/Bax ratio was significantly increased in the AQP8 overexpression group, whereas it was significantly decreased in the AQP8 knockdown group. This supports the idea that the effect of AQP8 on apoptosis in glioma cells is related to the Bcl-2/Bax ratio. Consistent findings were obtained in the analysis of the intracellular redox state.

Prata et al. [28] discovered that sulforaphane could regulate the expression of AQP8 in leukemia cells, thereby affecting the expression of Nox-2, an intracellular redox-related protein, and ultimately affect the growth of leukemia cells. However, they did not investigate the influence of changes in AQP8 expression on the intracellular redox level, and sulforaphane is not a specific AQP8-regulating substance. To our best knowledge, our study is the first to report that changes in AQP8 expression can affect not only ROS production but also the levels of GSH, MDA, and SOD. ROS is a key influencing factor of redox levels in glioma cells, whereas GSH, MDA, and SOD are key reducing enzymes. These results suggest that increased AQP8 expression can protect glioma cells from higher redox levels by improving redox enzyme activity and, to a certain extent, by increasing ROS levels, which is conducive to glioma cell proliferation and growth.

PTEN and AKT are downstream regulatory proteins of ROS. Measurements of PTEN, AKT, and p-AKT protein levels in glioma cells showed that PTEN expression was decreased as AQP8 expression was increased. Meanwhile, p-AKT expression was increased, while cell proliferation was accelerated. AQP8 knockdown had the opposite effect. The changes in PTEN and AKT phosphorylation levels were consistent with those reported in previous studies [29–31]. PTEN inhibited the phosphorylation of AKT in glioma cells by antagonizing the activity of tyrosine kinase and affected the proliferation of tumor cells, thus playing a tumor suppressor role. These results indicate that AQP8 may positively affect the new redox balance state in cells by mediating the ROS signaling pathway and promoting the proliferation and growth of glioma cells.

However, it was reported that AQP8 expression is lower in colon cancer tissues than in normal colon tissues [32]. AQP8 overexpression in the colorectal cancer cell lines SW480 and HT-29 inhibited the growth and invasion of colorectal cancer cells by inactivating the PI3K/AKT signaling pathway and inhibiting PCDH7 expression [33]. To fully understand the expression and function of AQP8 in other tumor tissues, the Clinical Proteomic Tumor Analysis Consortium (CPTAC), International Cancer Proteogenome Consortium (ICPC), and The Cancer Genome Atlas (TCGA) databases were searched, and we found no association between AQP8 and a wide range of tumors. However, we found some correlation between AQP8 mRNA and extensive tumors in the tumor immune evaluation resources (TIMER) database (https://cistrome.shinyapps.io/TIMER/)[34]. The results showed that the AQP8 is mostly expressed in tumor tissues rather than normal tissues. The data also showed that AQP8 expression was significantly downregulated in colorectal adenocarcinoma, rectal adenocarcinoma, and UCEC (uterine endometrial carcinoma), but significantly upregulated in renal clear cell carcinoma, hepatocellular carcinoma, and thyroid carcinoma compared with normal tissues. This database also showed that the expression of AQP8 in glioma was not high, but there was no control. This is inconsistent with the results we found in human gliomas samples before. The reason for our analysis to affect the sequencing results may be that the final sequencing expression values were different due to the different sequencing time and depth of the samples. In any case, the data are suggesting that AQP8 may play different roles in different tumor types, thus warranting further research.

Nevertheless, this study did not further expand on these aspects to prove the capability of AQP8 in promoting glioma proliferation by including extensive animal experiments, patient-derived model experiments and other techniques, and the response of other AQP family members with the extension of time after AQP8 knockdown is unknown. It is still a long way from clarifying the exact mechanism of action of AQP8 in the development of glioma and using AQP8 as a therapeutic target for glioma, which also makes the results of this paper somewhat encouraging, but with big limitation at the same time.These studies will be conducted in the future.

## Conclusions

This is a preliminary suggestion that AQP8 overexpression can promote the proliferation, migration, and invasion of glioma cells, whereas AQP8 knockdown can induce their apoptosis and inhibit proliferation and growth. Increased AQP8 expression may promote glioma proliferation by mediating ROS levels, regulating the levels of PTEN/AKT and other downstream proteins, and forming a new redox equilibrium state in cells. Thus, AQP8 may be a potential therapeutic target in glioma.

Abbreviations.

AKT, serine/threonine protein kinase; AQP, aquaporin; DHE, dihydroethidium; DAPI, 4,6-diamino-2-phenylindole; DMEM, Dulbecco’s Modified Eagle Medium; EDU, 5-ethyl-2-deoxyuridine; EP, Eppendorf; FBS, fetal bovine serum; GSH-PX, glutathione peroxidase; IL, interleukin; MDA, malondialdehyde; PAGE, polyacrylamide gel electrophoresis; p-AKT, phosphorylated AKT; PBS, phosphate-buffered saline; PCR, polymerase chain reaction; PTEN, phosphatase and tensin homolog; PVDF, polyvinylidene fluoride; ROS, reactive oxygen species; SDS, sodium dodecyl sulfate; SOD, superoxide dismutase; WB, western blotting.

## Electronic supplementary material

Below is the link to the electronic supplementary material.

Supplementary Material 1

Supplementary Material 2

Supplementary Material 3

Supplementary Material 4

Supplementary Material 5

## Data Availability

The datasets generated during and during the current study are not publicly available because parts of the data were designed for subsequent experiments; however, the datasets are available from the corresponding author upon reasonable request.
