# Supplementary material for: AQP8 promotes glioma proliferation and growth, possibly through the ROS/PTEN/AKT signaling pathway
Source: BMC Cancer. 2023 Jun 6;23:516. doi: 10.1186/s12885-023-11025-8 (PMC10242804; doi:10.1186/s12885-023-11025-8)

1.Exposure conditions： Use Image Lab software for automatic exposure, no artificial adjustment to the exposure value.

2.The whole membrane Information：

A172：

Maker	Protein	Merge

PTEN protein

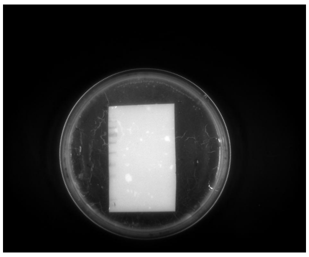
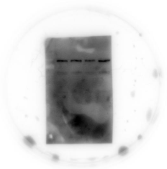
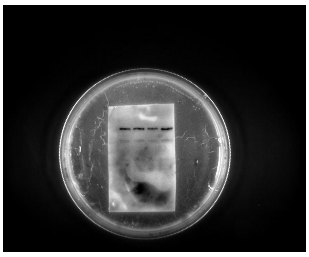

AKT protein

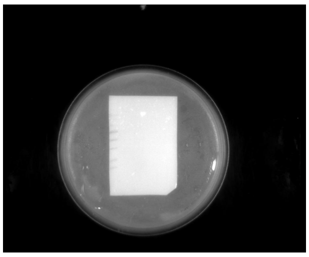
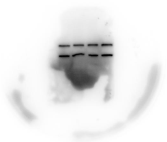
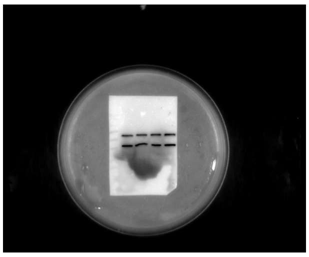

Bcl-2 protein

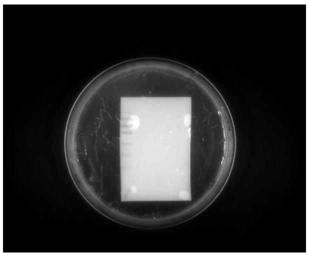
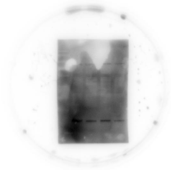
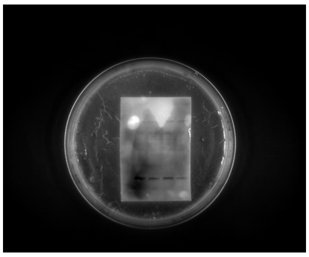

Bax protein

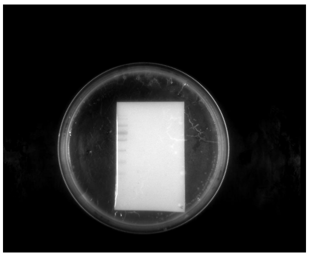
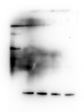
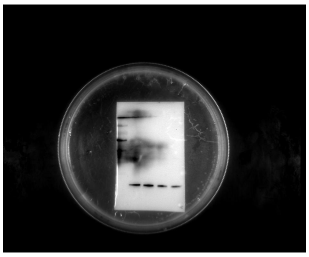

P-AKT protein

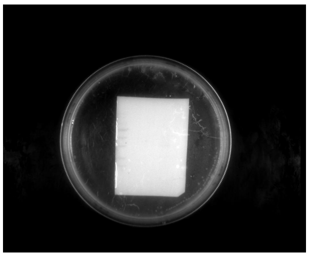
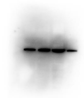
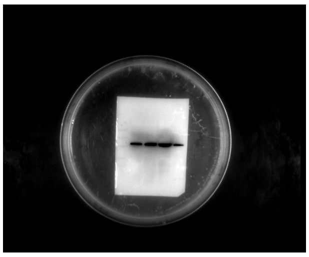

AQP8 protein

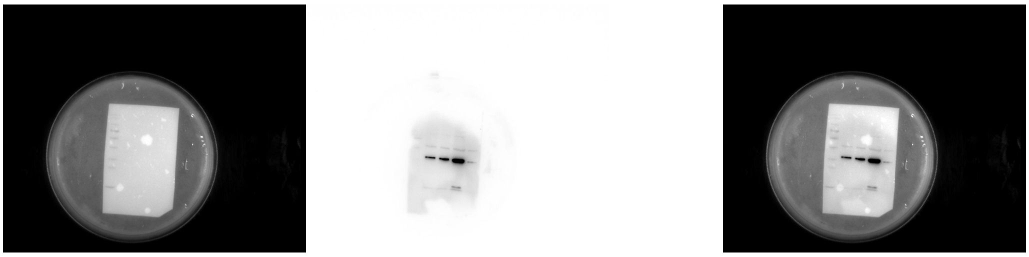

β -actin protein

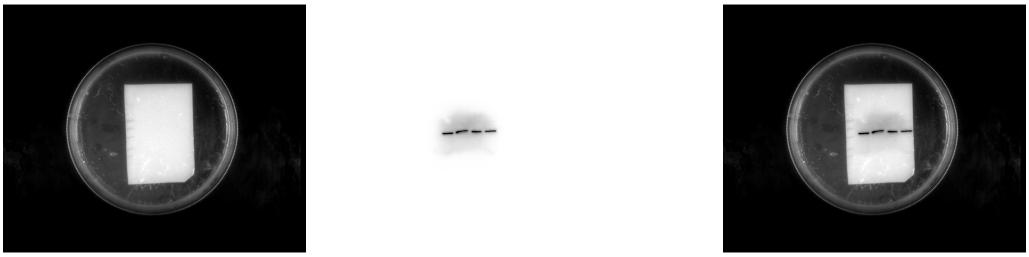

U251：

PTEN protein

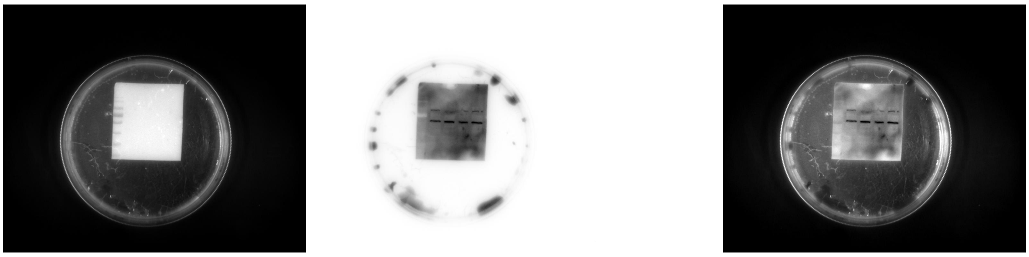

AKT protein

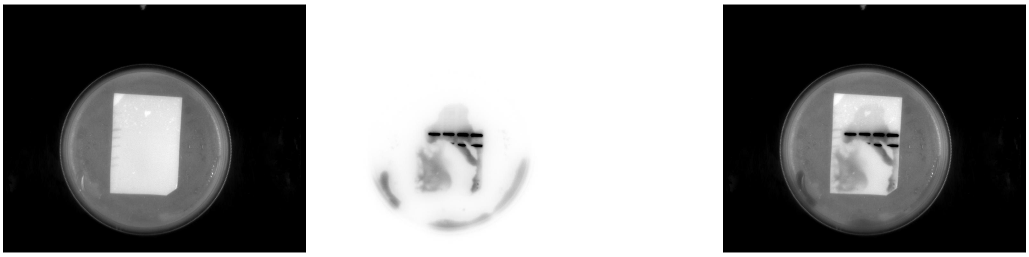

P-AKT protein

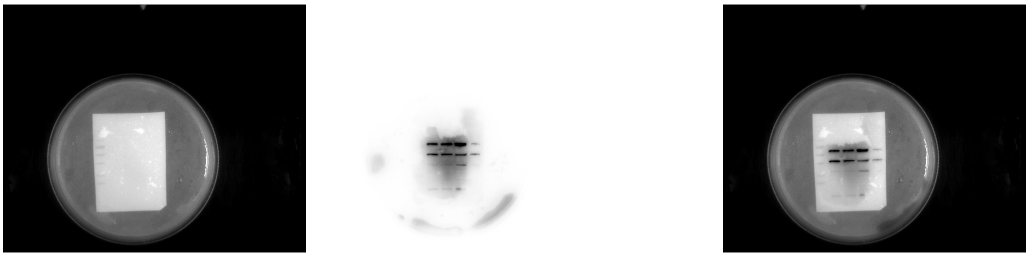

Bcl-2 protein

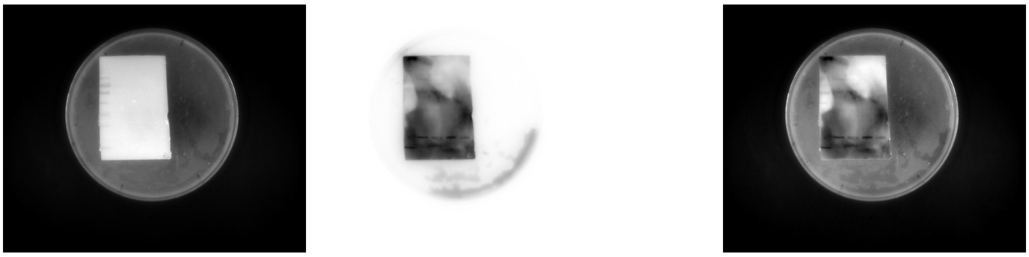

Bax protein

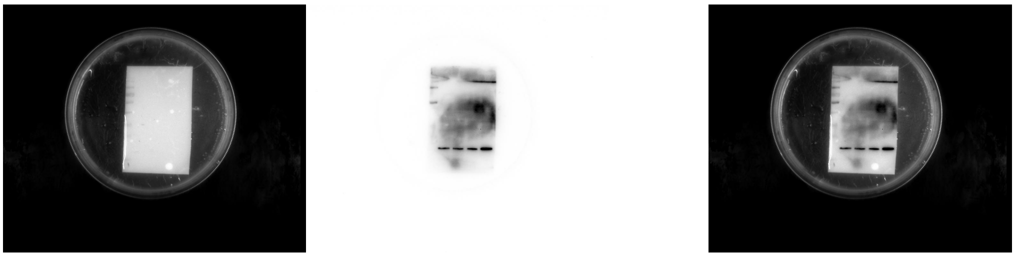

AQP8 protein

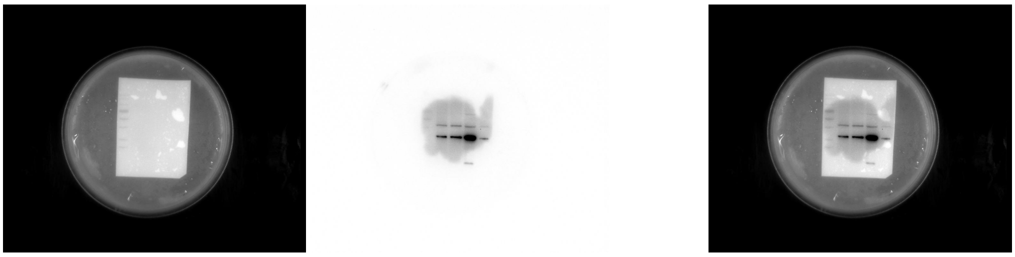

β -actin protein

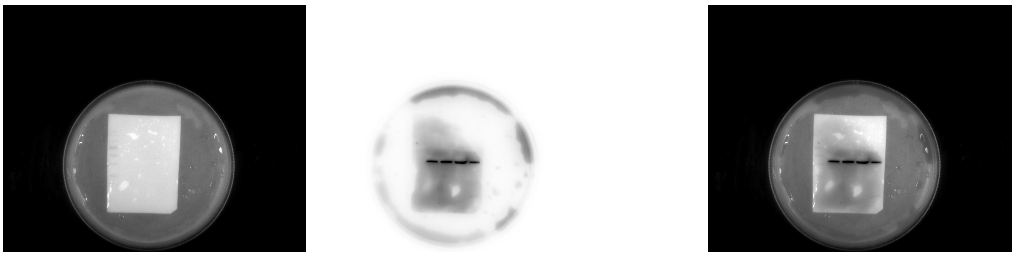

Supplement: Supplementary file 1 — Supplementary Material 1 [file 12885_2023_11025_MOESM1_ESM.pdf]
