# Supplementary material for: AQP8 promotes glioma proliferation and growth, possibly through the ROS/PTEN/AKT signaling pathway
Source: BMC Cancer. 2023 Jun 6;23:516. doi: 10.1186/s12885-023-11025-8 (PMC10242804; doi:10.1186/s12885-023-11025-8)

Supplemental information

WB：expression of AQP8 in U87, A172 and U251 cell lines

1. original imagines of all blots for AQP8 on Fig1A in the manuscript:

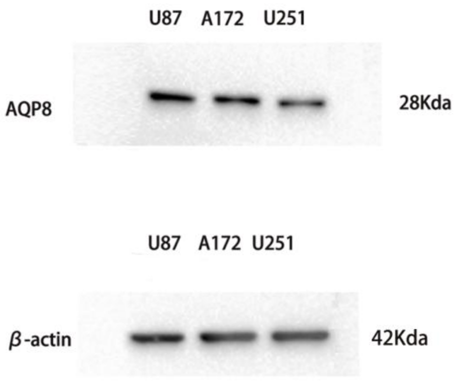

2. WB replicate:

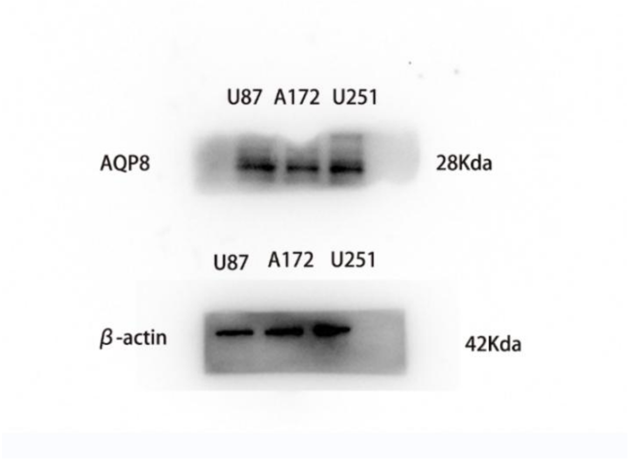

3. WB replicate:

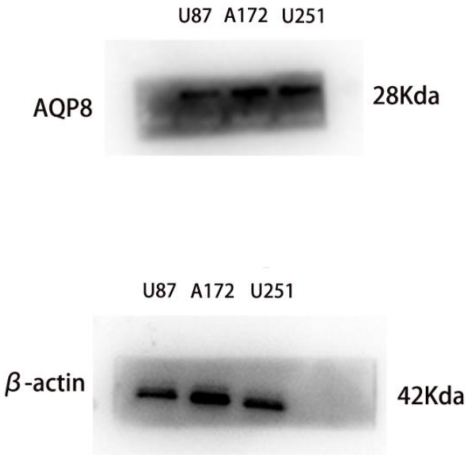

qPCR: amplification and melt curves of qPCR for AQP8 mRNA in all groups of A172 and U251, were divided into: a control group, a negative control group, three overexpression groups, three knock-down groups.

1. A172:

1.1 amplification curve of AQP8 mRNA:

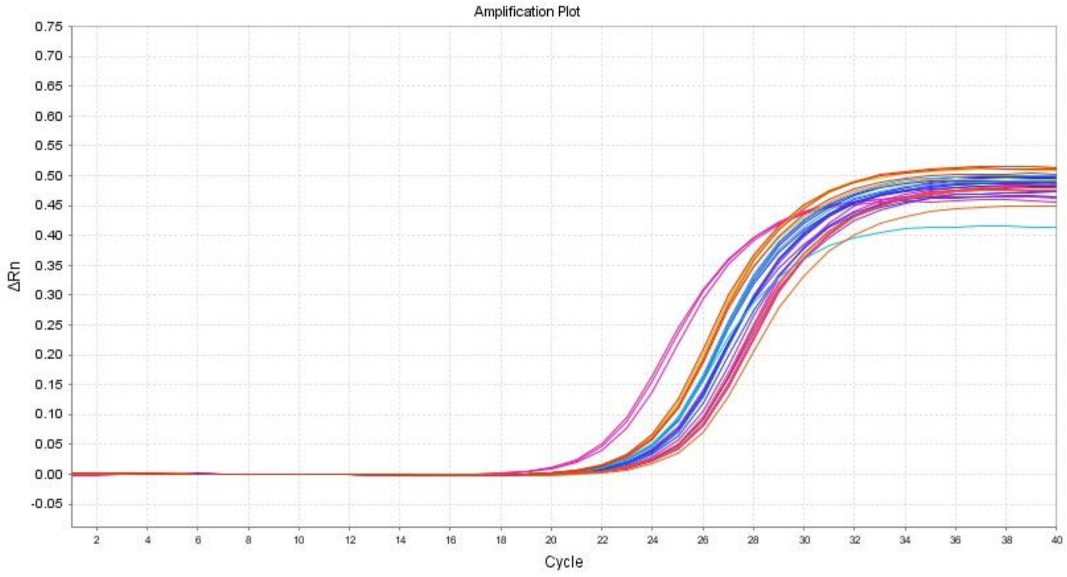

1.2 amplification curve of β-actin mRNA:

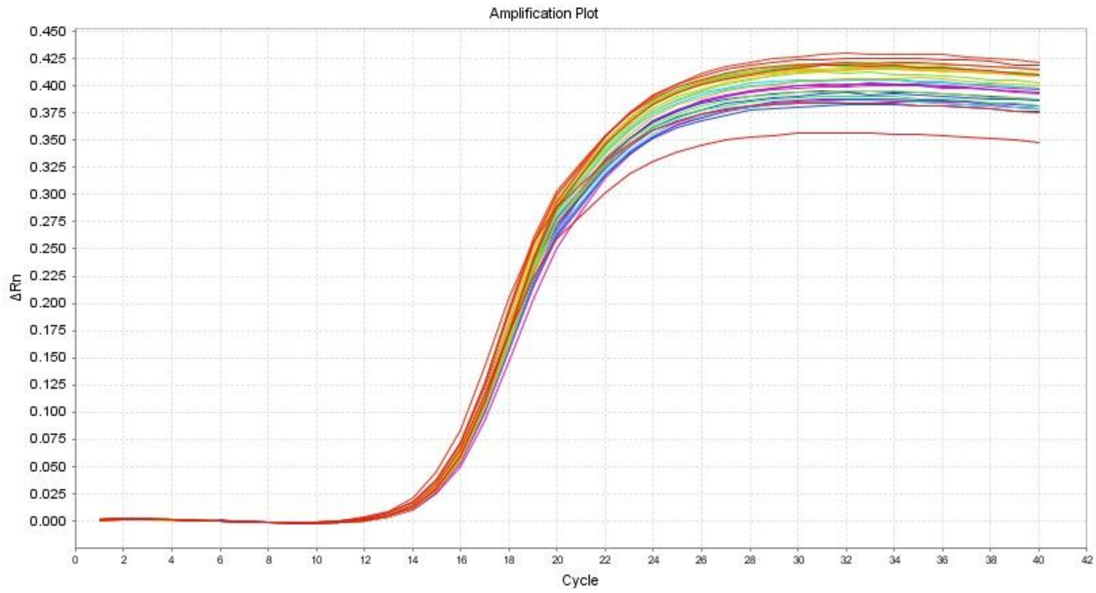

1.3 melt curve of AQP8 mRNA :

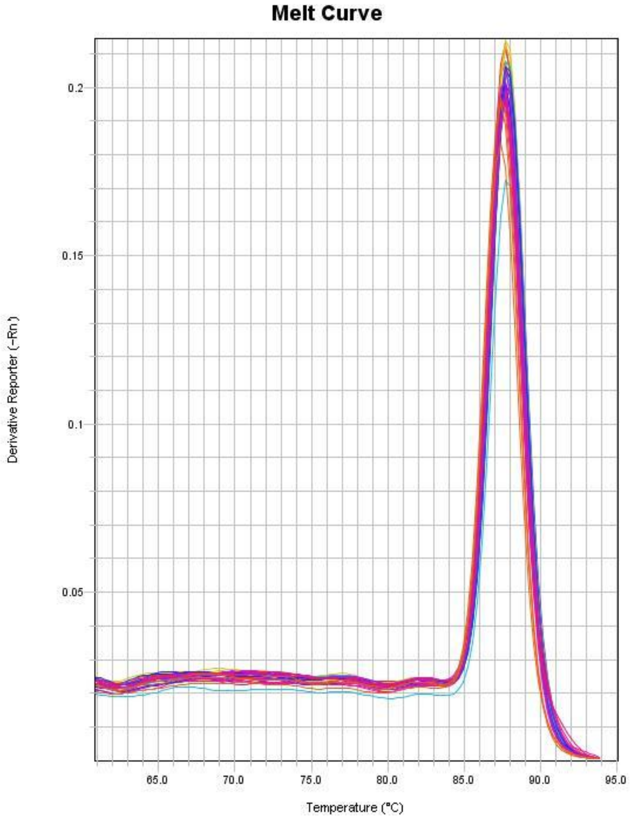

1.4 melt curve of β-actin mRNA :

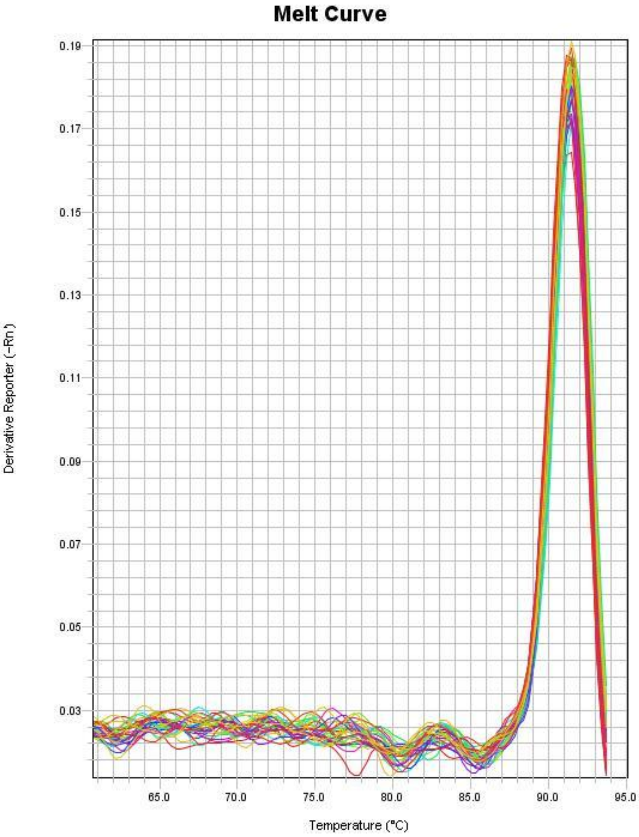

2. U251:

2.1 amplification curve of AQP8 mRNA:

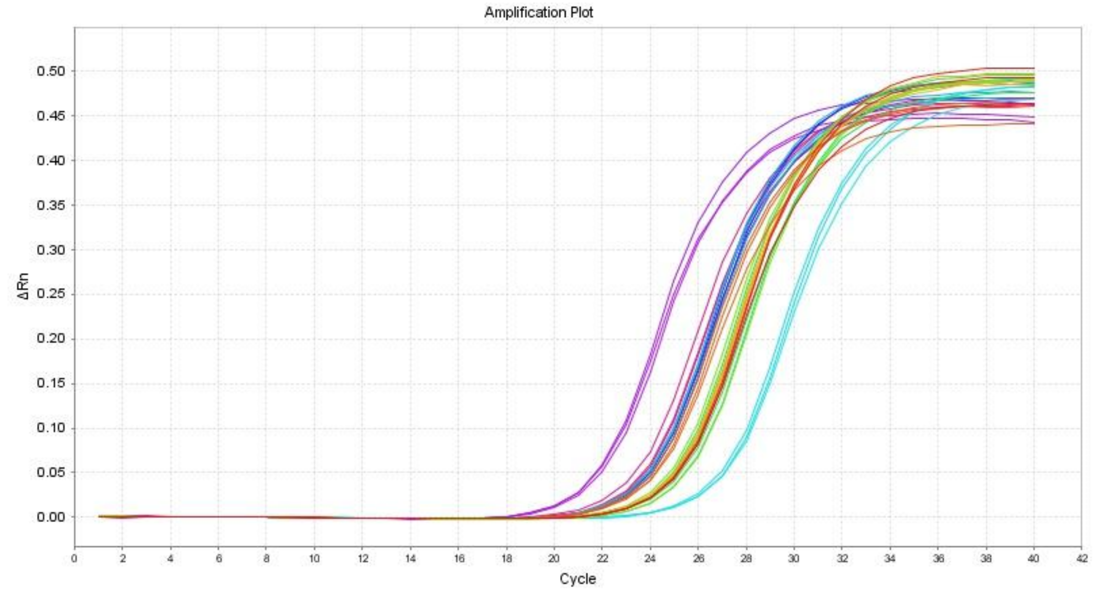

2.2 amplification curve of β-actin mRNA:

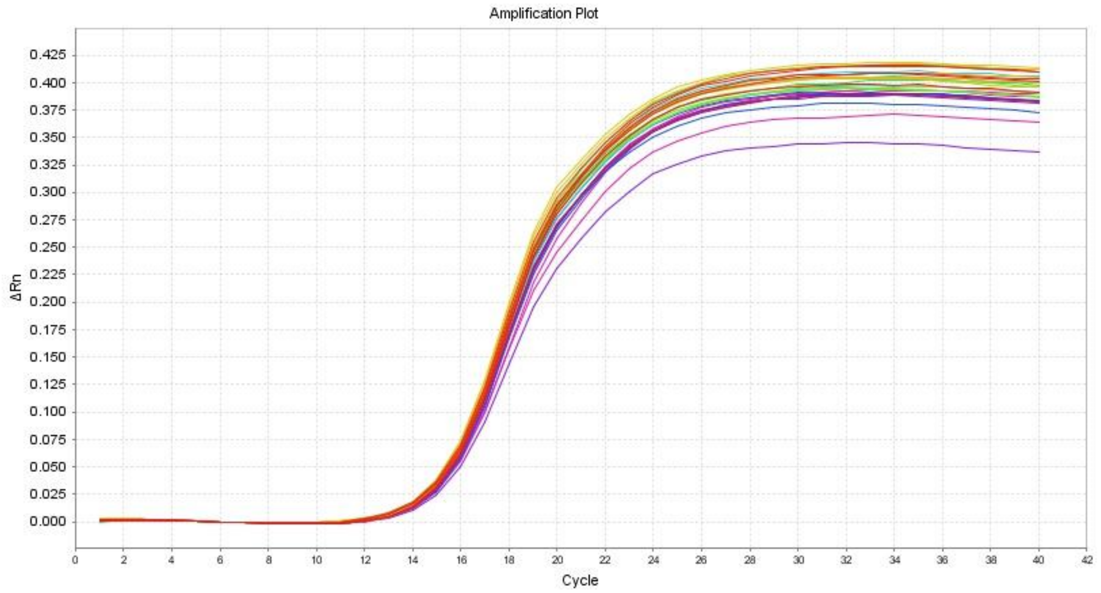

2.3 melt curve of AQP8 mRNA:

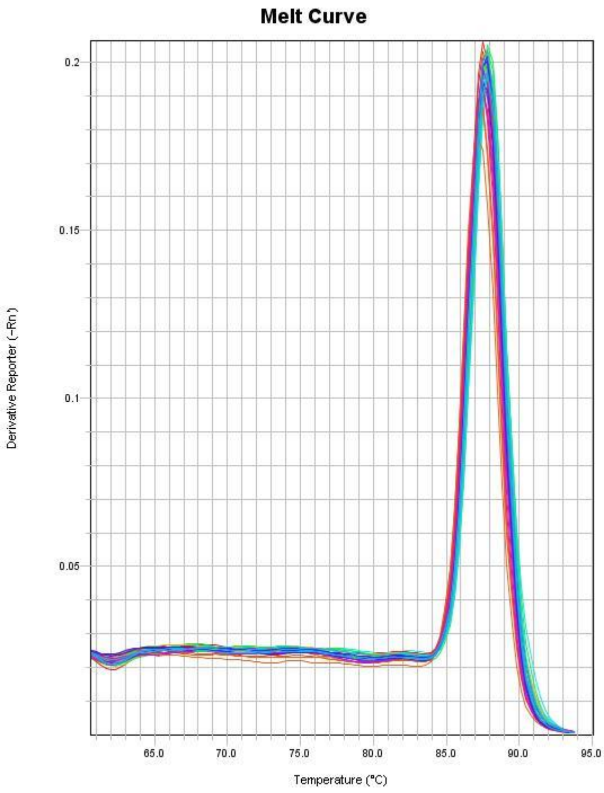

2.4 melt curve of β-actin mRNA:

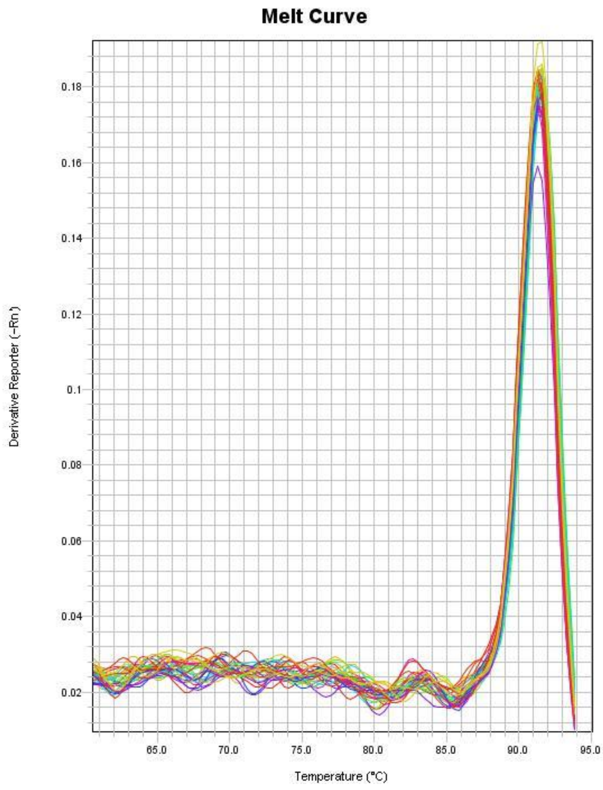

Supplement: Supplementary file 2 — Supplementary Material 2 [file 12885_2023_11025_MOESM2_ESM.pdf]
