# Supplementary material for: AQP8 promotes glioma proliferation and growth, possibly through the ROS/PTEN/AKT signaling pathway
Source: BMC Cancer. 2023 Jun 6;23:516. doi: 10.1186/s12885-023-11025-8 (PMC10242804; doi:10.1186/s12885-023-11025-8)
Supplement: Supplementary file 3 — Supplementary Material 3 [file 12885_2023_11025_MOESM3_ESM.pdf]

Supplemental materials

All original imagines of all blots, with full length, membrane boundary visible for Fig1E,F and Fig6A,D,F,I in the manuscript as below.

Fig1-E:

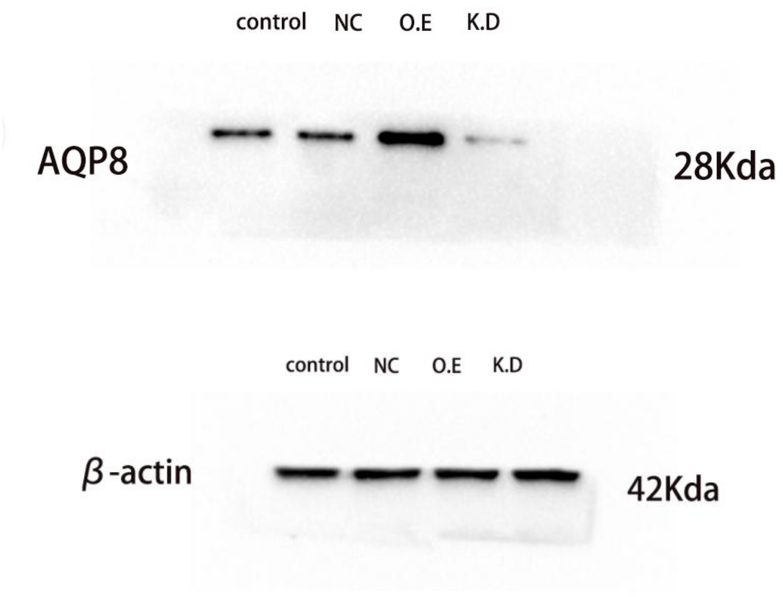

Fig1-F:

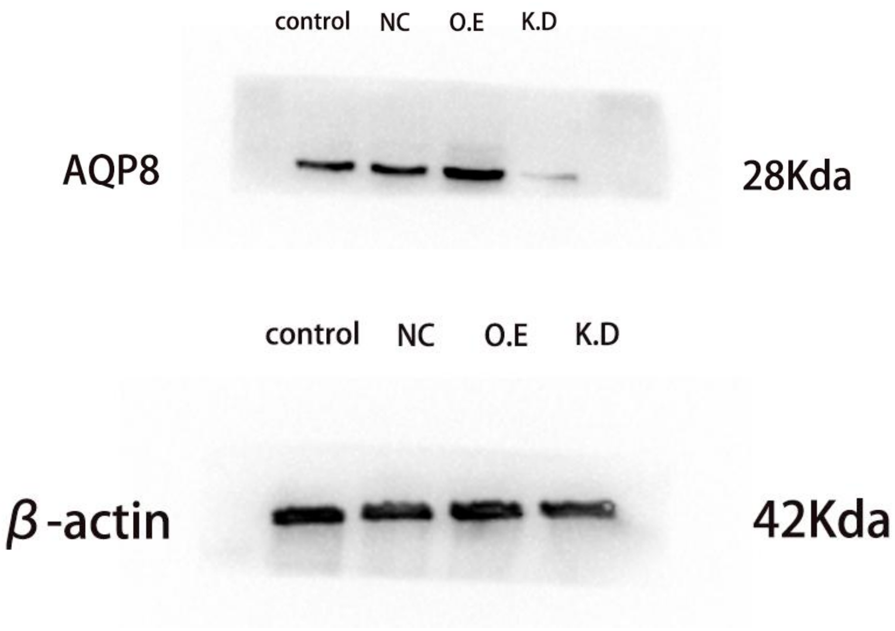

Fig6-A:

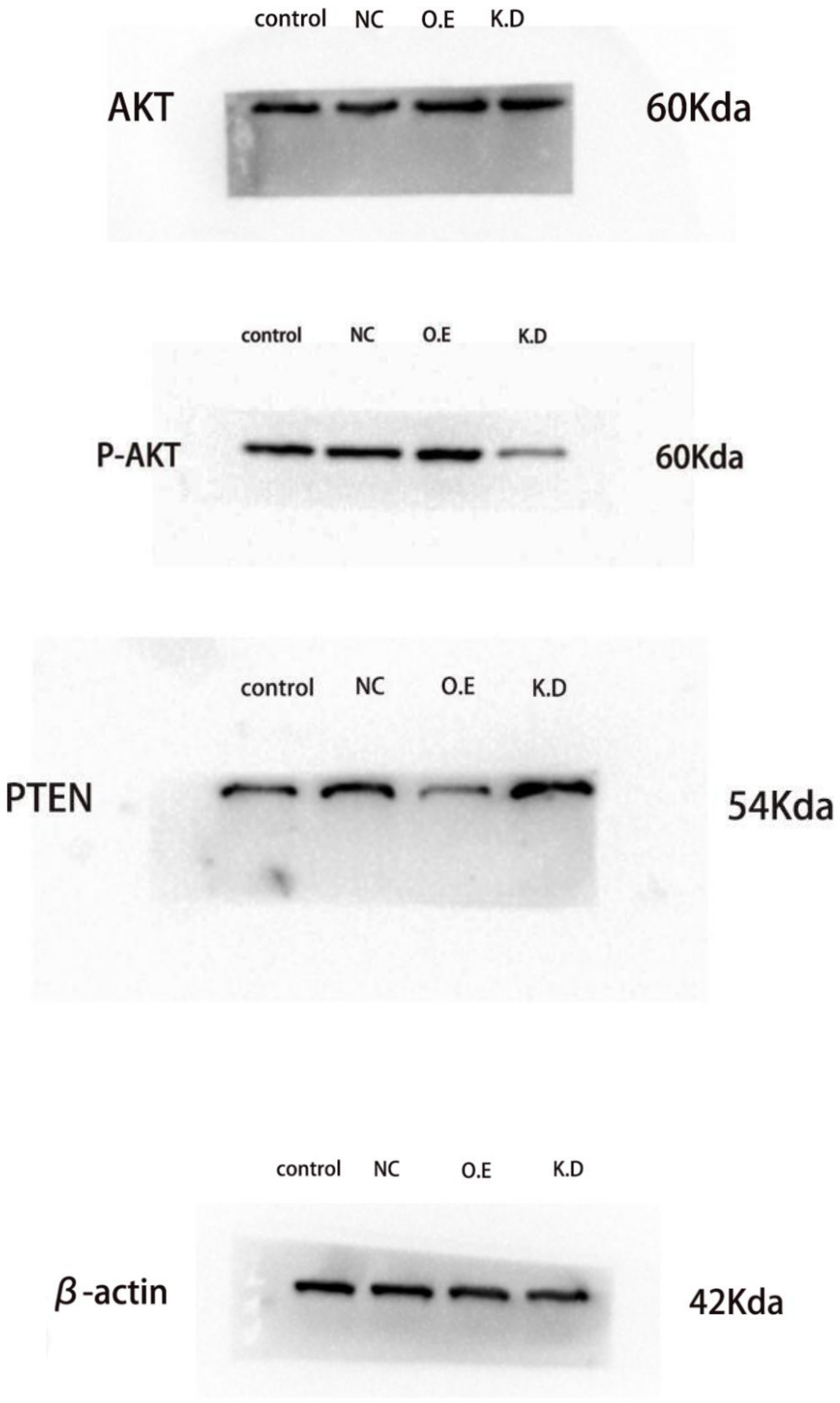
control
NC
O.E
K.D
AKT
60Kda
control
NC
O.E
K.D
P-AKT
60Kda
control
NC
O.E
K.D
PTEN
54Kda
control
NC
O.E
K.D
β-actin
42Kda

Fig6-D:

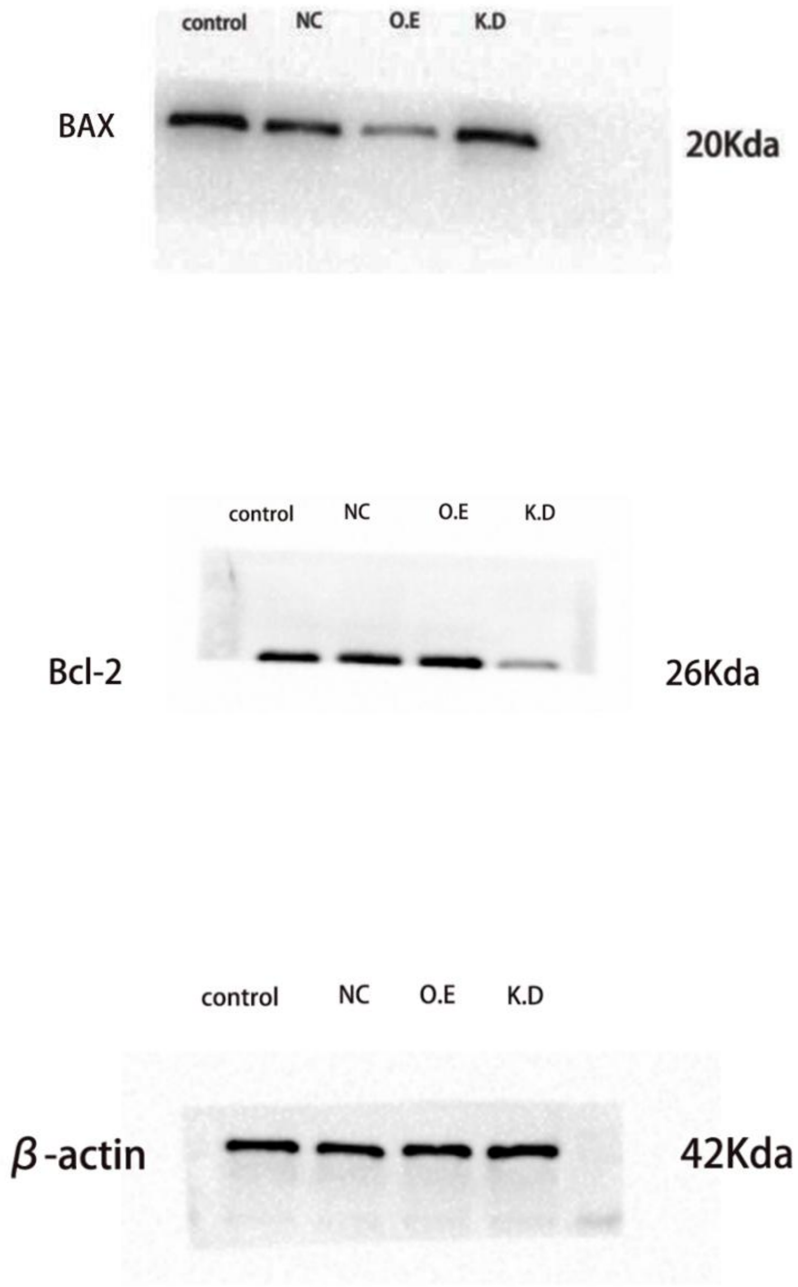
control
NC
O.E
K.D
BAX
20Kda
control
NC
O.E
K.D
Bcl-2
26Kda
control
NC
O.E
K.D
β-actin
42Kda

Fig6-F:

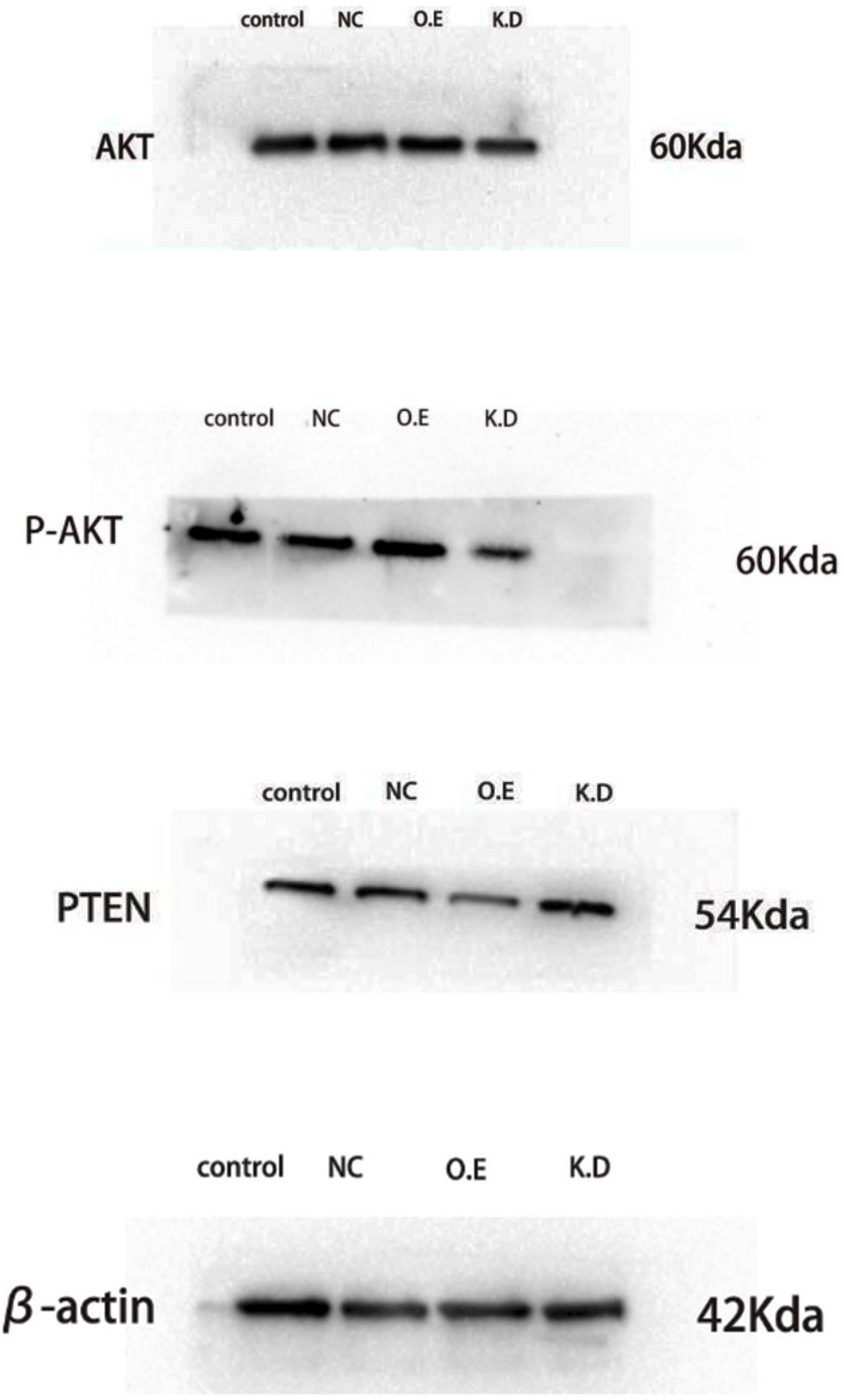
control
NC
O.E
K.D
AKT
60Kda
control
NC
O.E
K.D
P-AKT
60Kda
control
NC
O.E
K.D
PTEN
54Kda
control
NC
O.E
K.D
β-actin
42Kda

Fig6-I:

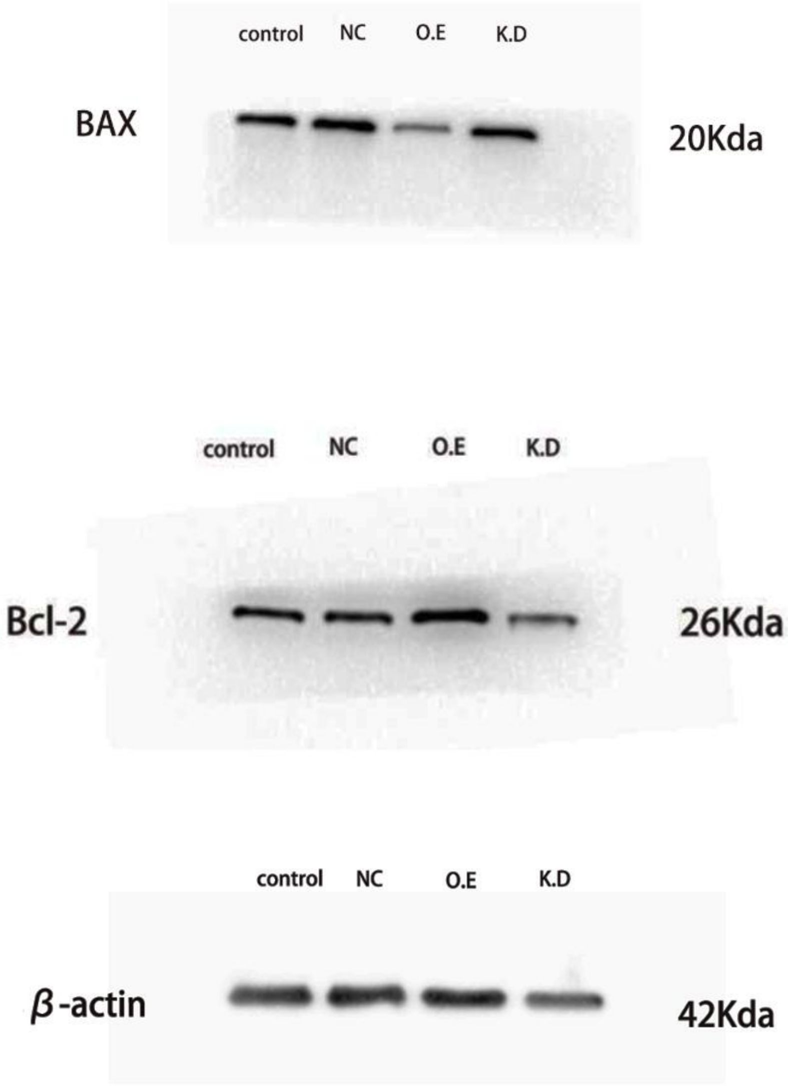

control
NC
O.E
K.D
BAX
20Kda
control
NC
O.E
K.D
Bcl-2
26Kda
control
NC
O.E
K.D
β-actin
42Kda
