# Supplementary material for: AQP8 promotes glioma proliferation and growth, possibly through the ROS/PTEN/AKT signaling pathway
Source: BMC Cancer. 2023 Jun 6;23:516. doi: 10.1186/s12885-023-11025-8 (PMC10242804; doi:10.1186/s12885-023-11025-8)

Supplemental information

Another original imagines of all blots, with full length, membrane boundary visible for each antibody which confirms specific detection of the target antigen in the manuscript show as below.

AQP8 in A172 cells:

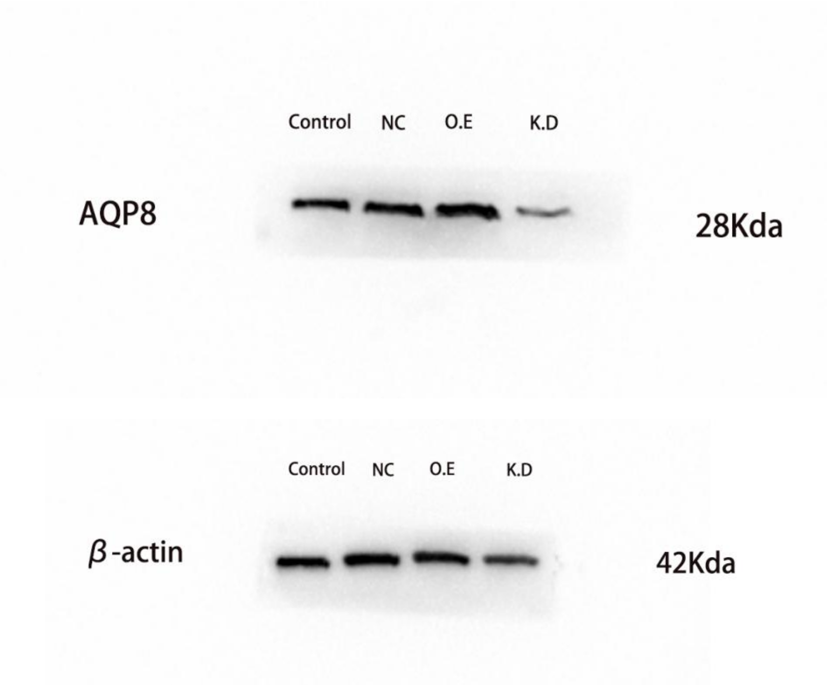

AQP8 in U251 cells:

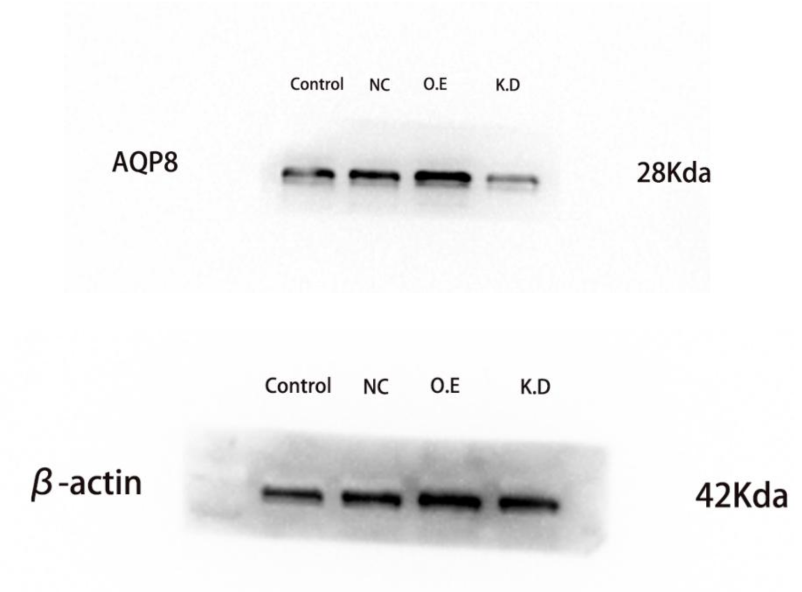

AKT, p-AKT, PTEN in A172 cells:

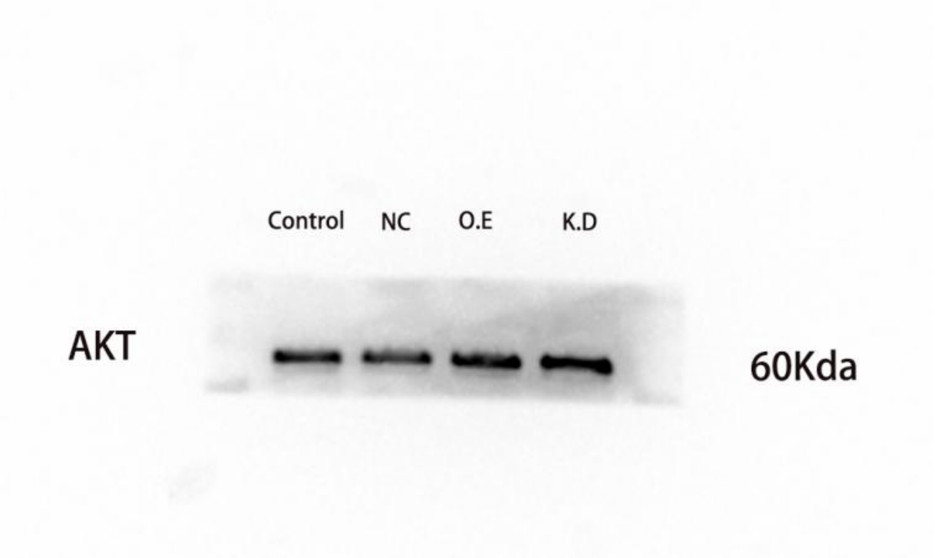

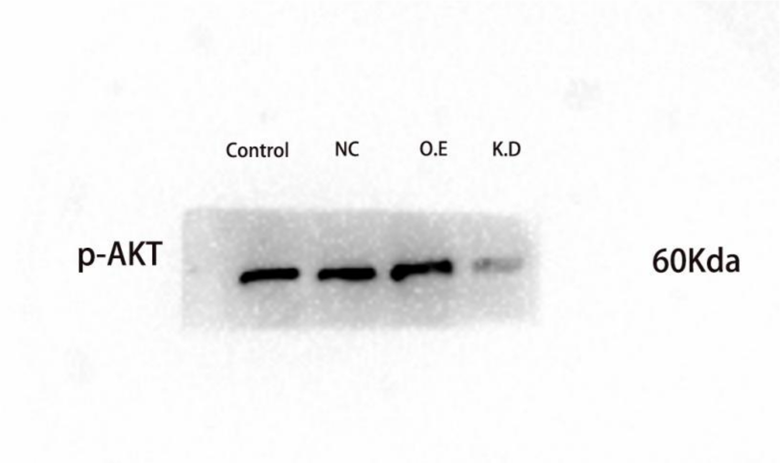

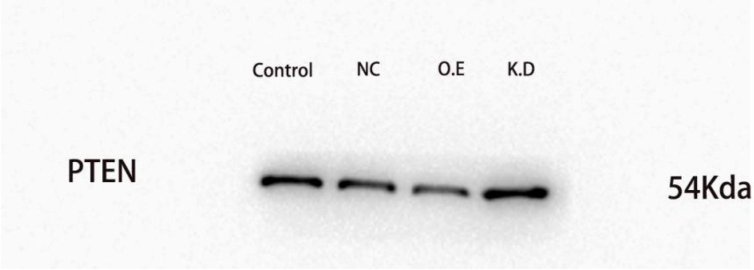

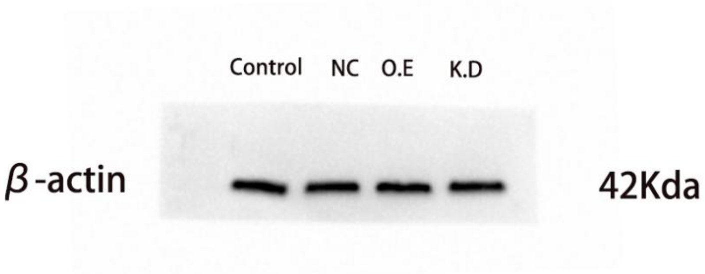

BAX, Bcl-2 in A172 cells:

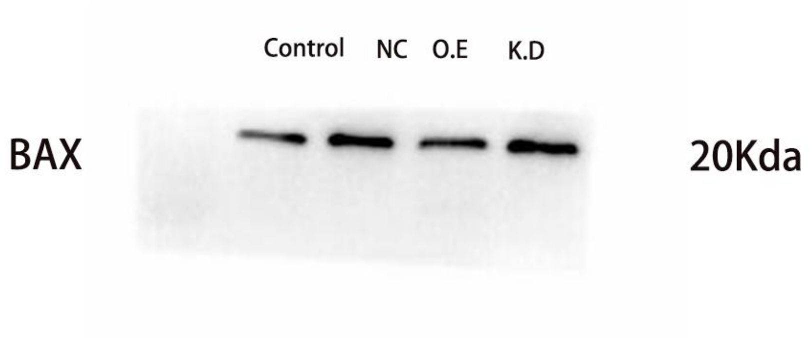

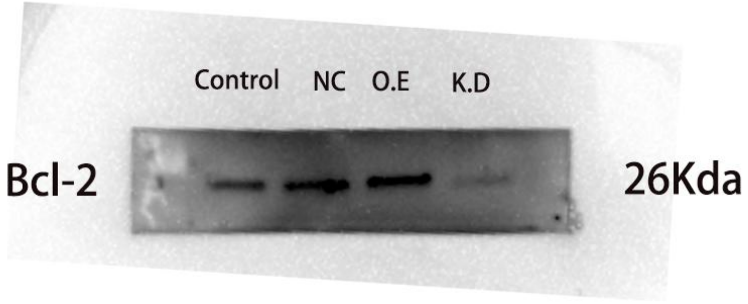

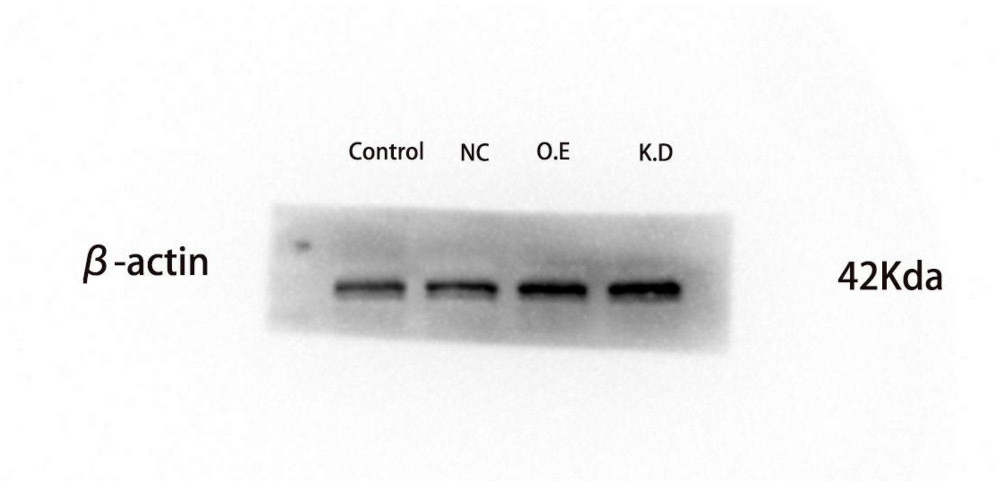

AKT, p-AKT, PTEN in U251 cells:

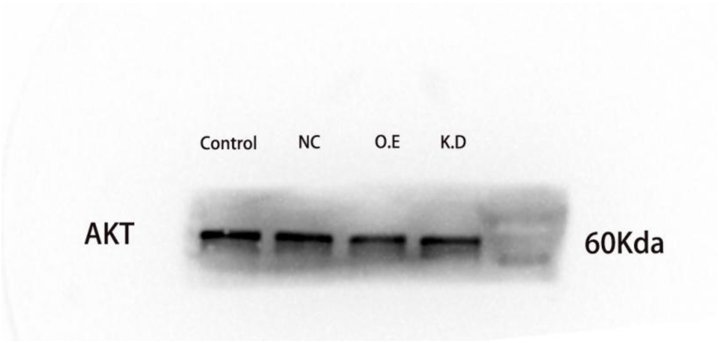

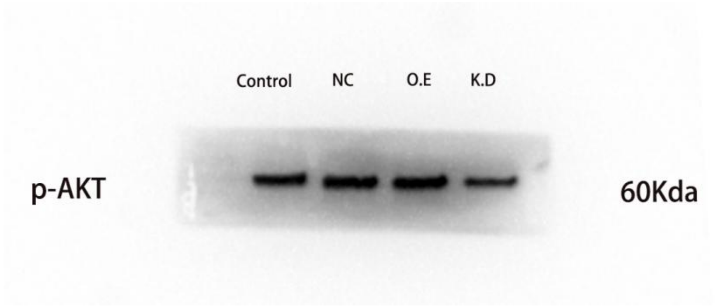

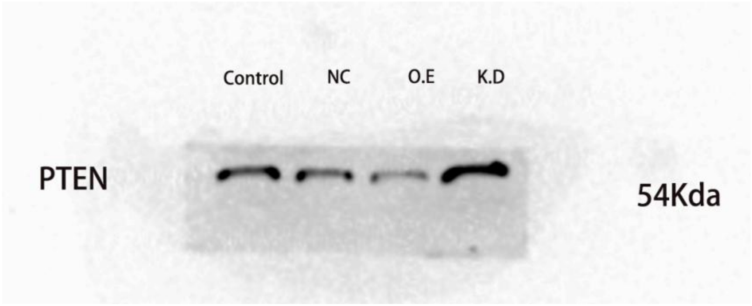

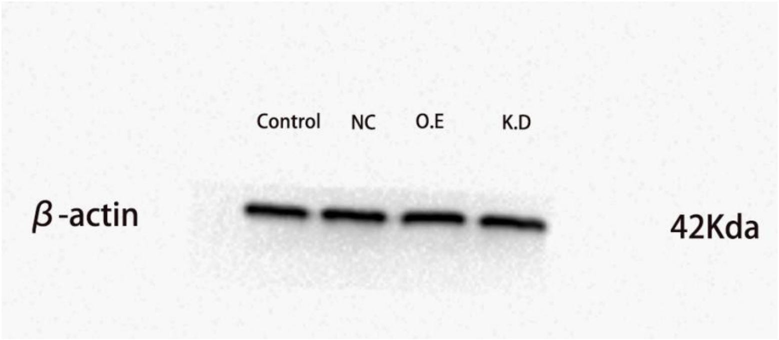

BAX, Bcl-2 in U251 cells:

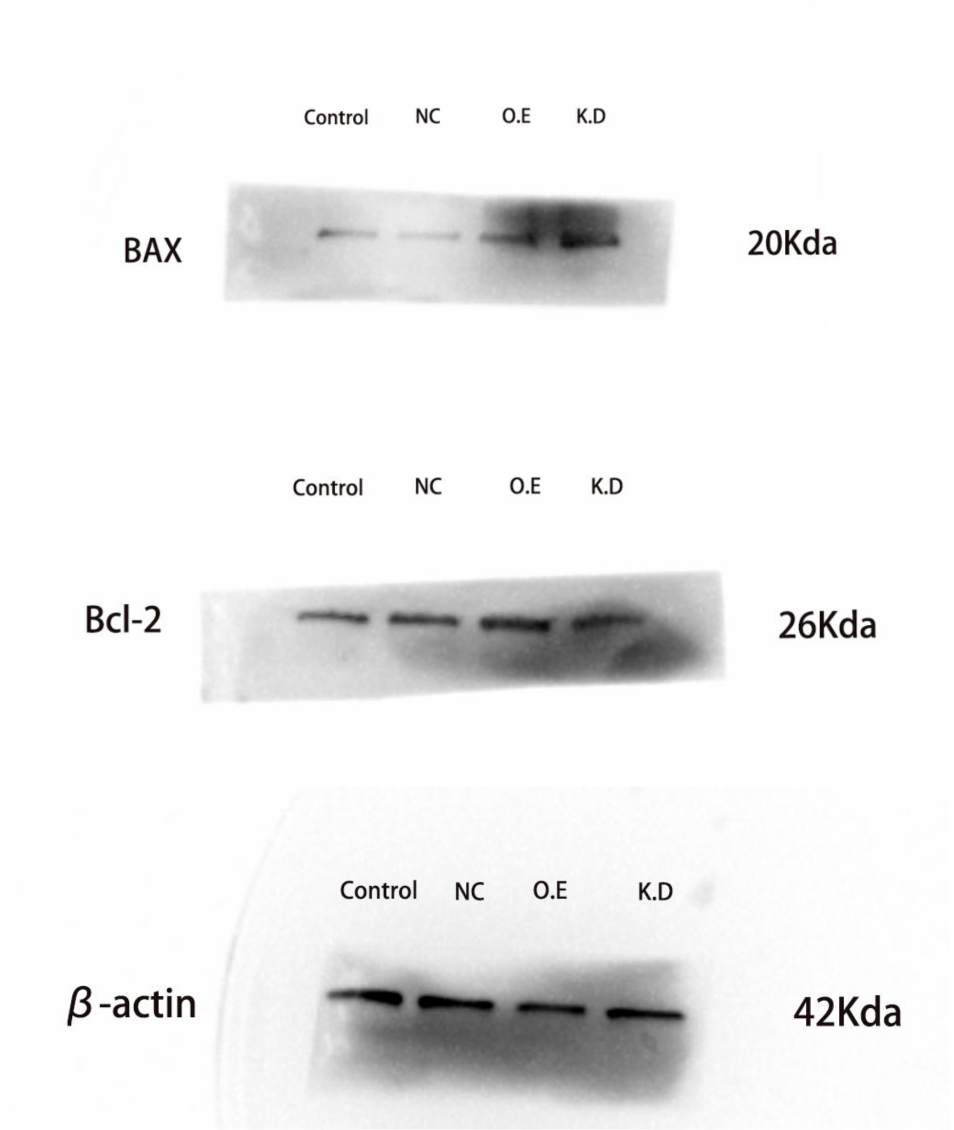

Supplement: Supplementary file 4 — Supplementary Material 4 [file 12885_2023_11025_MOESM4_ESM.pdf]
