# Supplementary material for: AQP8 promotes glioma proliferation and growth, possibly through the ROS/PTEN/AKT signaling pathway
Source: BMC Cancer. 2023 Jun 6;23:516. doi: 10.1186/s12885-023-11025-8 (PMC10242804; doi:10.1186/s12885-023-11025-8)

Supplemental materials

We added a negative control group (NC1), which is the control for over-expression, using empty vector virus. The cell viability of each group was detected by CCK-8 and MTT.

Details of supplemental experiments as follows：

1. Methods

1.1 Experimental groups

Glioma cells A172 and U251 were used as the experimental subjects. The experiment was divided into five groups, namely: Normal glioma cells (Control), control group 1 infected with empty vector virus (negative control, NC1), control group 2 infected with nontargeting gRNA virus (negative control, NC2), The over-expression group (O.E) infected with the overexpression virus and the knock-down group (K.D) infected with the knockdown virus. As indicated in the manuscript, the viruses both K.D and O.E group continued to be used for the supplementary experiment.

1.2 WB and RT-qPCR

Previously described in detail, located in the section of methods in manuscript.

1.3 MTT（3-(4,5)-dimethylthiahiazo (-z-y1)-3,5-di- phenytetrazoliumromide, MTT）

A172 and U251 cells were inoculated into 96-well plates with 5×10^3 cells per well. After incubation for 24 h, 10 μl 5mg /ml MTT solution (Sigma, USA) was added to each well, incubated at 37°C for 4 h and discarded. 100 μl dimethyl sulfoxide (DMSO, Sigma) was added to each well. Shake the orifice plate at room temperature at a low speed for 15 minutes to completely dissolve the purple precipitate. The absorbance value of each well was measured at OD490 nm.

1.4 CCK-8(Cell Counting Kit-8,CCK-8）

Cell suspensions of A172 and U251 were inoculated into the bottom of the 96-well plate with 100ul per well, so that the number of cells in each well was 5×10^3. The culture plates were placed in the incubator for a period of time. After the cells were fully adherent to the wall, CCK8 solution (10 μl) was added into each well every 24 h for 120 h. After incubating at 37°C for 3 h, the absorbance of each well at 450nm was measured using microplate reader.

2. Results

2.1 Validation of viruses

RT-qPCR results showed that the mRNA expression of AQP8 in the O.E group was significantly higher than that in the NC1 group, and its mRNA expression in the K.D group was significantly lower than that in the NC2 group (Figure 1A,B). WB results showed that AQP8 protein expression in the O.E

group was significantly higher than that in the NC1 group, and its protein expression in the K.D group was significantly lower than that in the NC2 group (Figure 1C-F). This indicated that lentivirus was effective and could play the role of over-expression and knock-down in both A172 and U251 cells, which could be used for follow-up experiments.

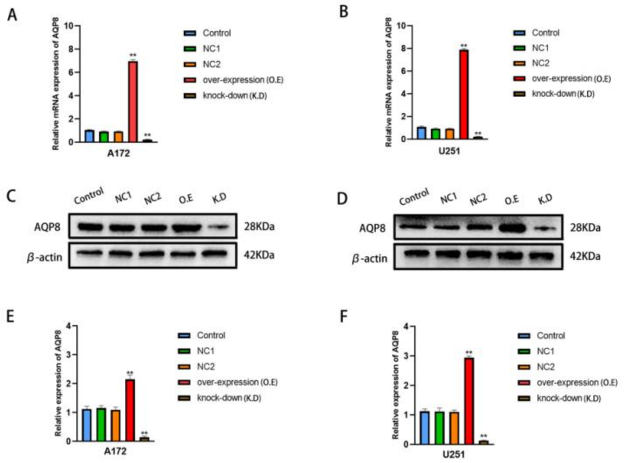

Fig. 1 Validation of viruses that overexpress and knockout AQP8. (A-B) Relative expression of AQP8 mRNA in A172 and U251 cells. (C-D) AQP8 protein expression in A172 and U251 cells. (E-F) Histogram analysis of AQP8 expression in A172 and U251 cells. Each group of cell detection sample N>4, The experiments were performed in triplicate (**P<0.01).

2.2 The effect of virus infection on cell viability

MTT and CCK-8 experiments showed that the proliferative activity of cells in the overexpression group was significantly stronger than that in the Control group, while that in the knockdown group was significantly weaker than that in the Control group (Figure2A-D). This indicates that increasing AQP8 expression in glioma cells to a certain extent is conducive to the proliferation of glioma cells, while decreasing AQP8 expression is not conducive to the proliferation of glioma cells.

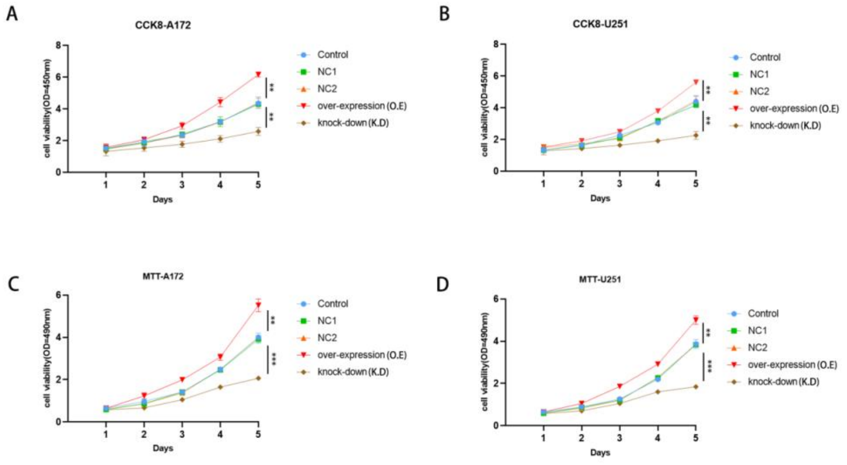

Fig. 2 Effect of AQP8 expression on the proliferation of glioma cells. The activity of A172 and U251 cells was evaluated by CCK-8 assay (A-B) and MTT assay(C-D). The detection time points were 24h, 48h, 72h, 96h and 120h after cell adhesion. Each group of cell detection sample N>4,The experiments were performed in triplicate (**P<0.01， ***P<0.001).

Repeated WB figures:

All original imagines of all blots, with full length, membrane boundary visible for each antibody which confirms specific detection of the target antigen in the manuscript as below.

AQP8 in A172 cells(1):

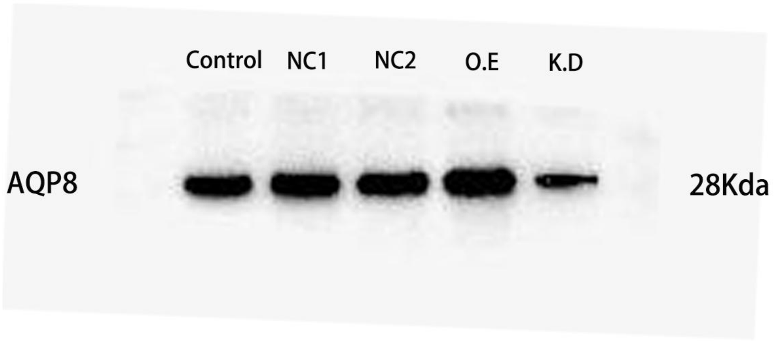

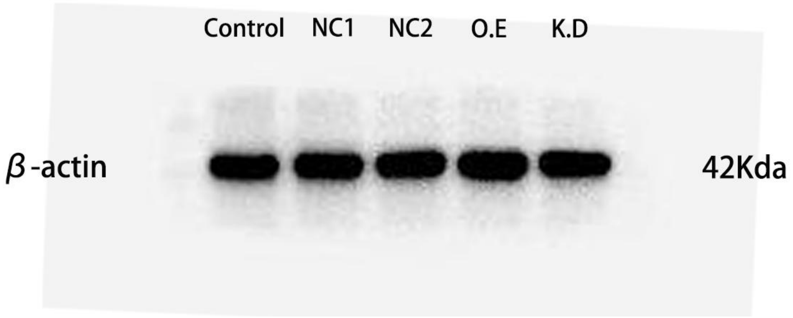

AQP8 in A172 cells(2):

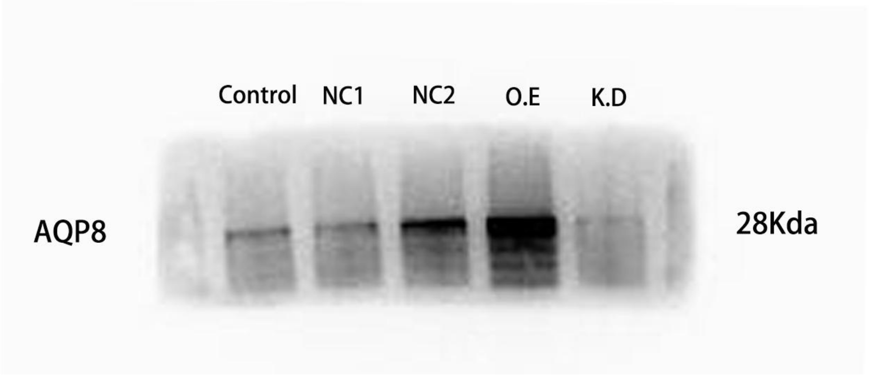

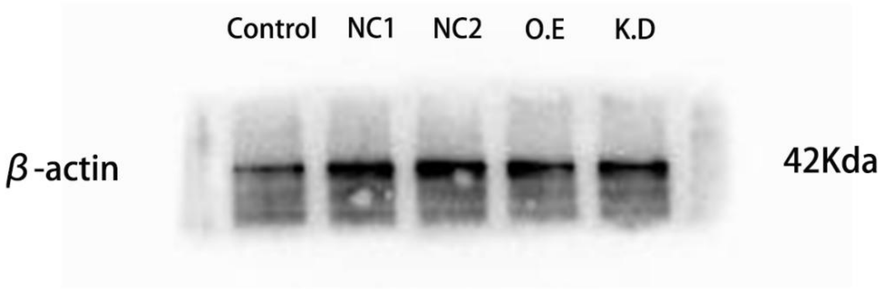

AQP8 in A172 cells(3):

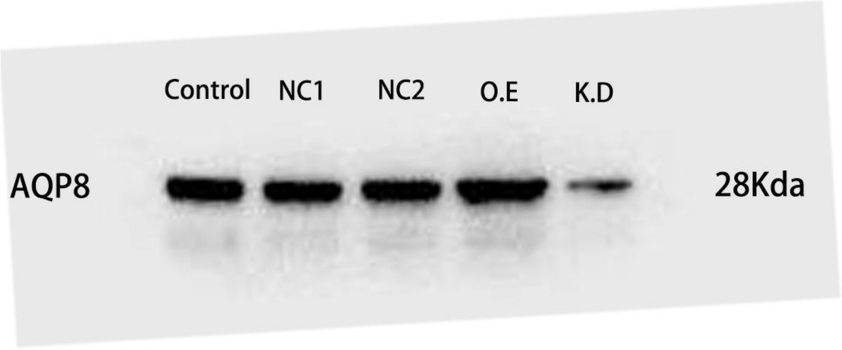

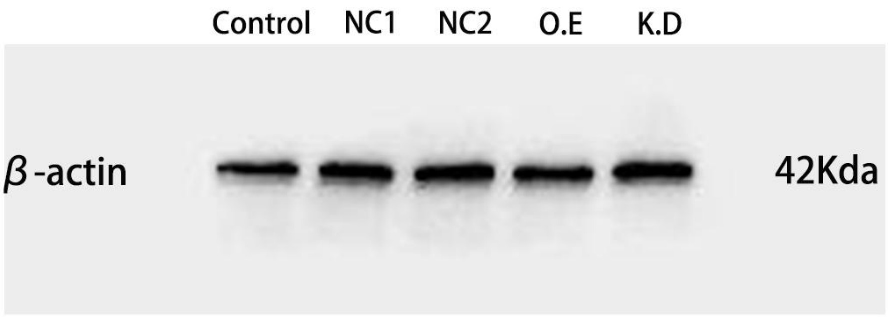

AQP8 in U251 cells(1):

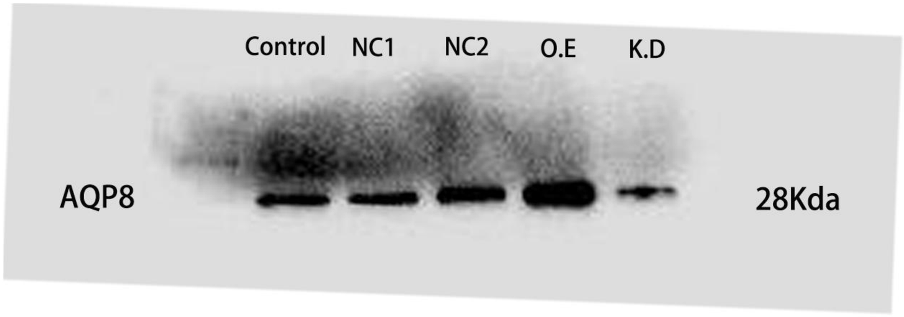

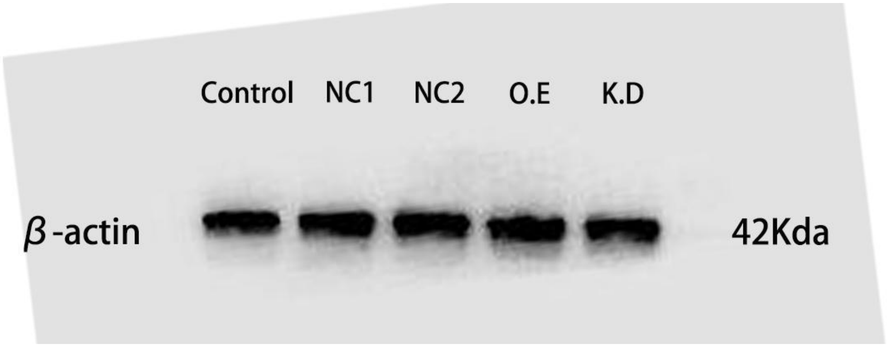

AQP8 in U251 cells(2):

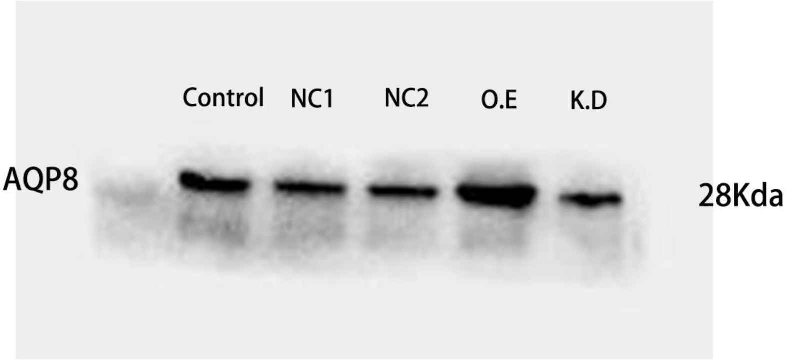

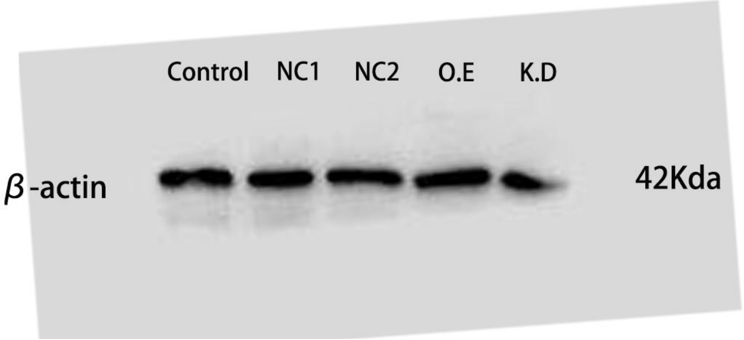

AQP8 in U251 cells(3):

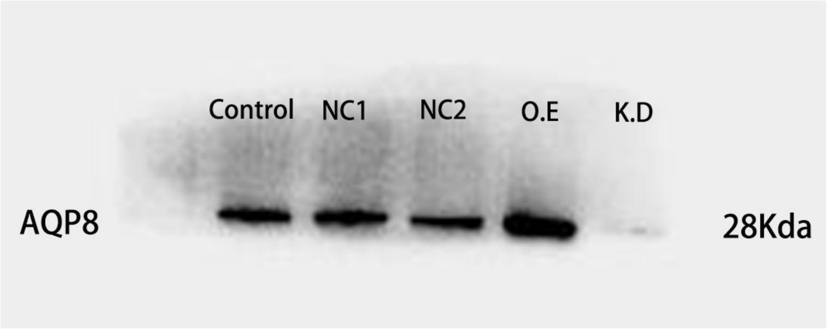

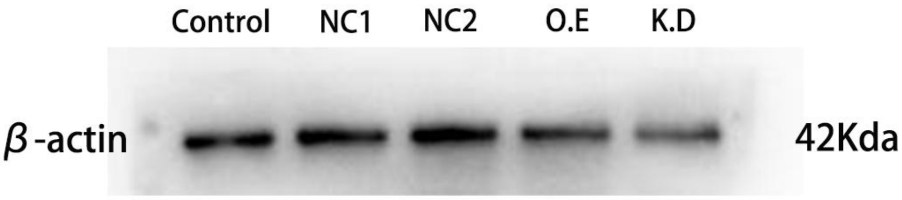

Supplement: Supplementary file 5 — Supplementary Material 5 [file 12885_2023_11025_MOESM5_ESM.pdf]
